# Transcriptome and Proteome Exploration to Model Translation Efficiency and Protein Stability in *Lactococcus lactis*

**DOI:** 10.1371/journal.pcbi.1000606

**Published:** 2009-12-18

**Authors:** Clémentine Dressaire, Christophe Gitton, Pascal Loubière, Véronique Monnet, Isabelle Queinnec, Muriel Cocaign-Bousquet

**Affiliations:** 1 Université de Toulouse; INSA, UPS, INP; LISBP, Toulouse, France; 2 INRA, UMR792 Ingénierie des Systèmes Biologiques et des Procédés, Toulouse, France; 3 CNRS, UMR5504, Toulouse, France; 4 INRA, Unité de Biochimie Bactérienne, UR477, Jouy en Josas, France; 5 Université de Toulouse; LAAS-CNRS, Toulouse, France; Max-Planck-Institute for Molecular Plant Physiology, Germany

## Abstract

This genome-scale study analysed the various parameters influencing protein levels in cells. To achieve this goal, the model bacterium *Lactococcus lactis* was grown at steady state in continuous cultures at different growth rates, and proteomic and transcriptomic data were thoroughly compared. Ratios of mRNA to protein were highly variable among proteins but also, for a given gene, between the different growth conditions. The modeling of cellular processes combined with a data fitting modeling approach allowed both translation efficiencies and degradation rates to be estimated for each protein in each growth condition. Estimated translational efficiencies and degradation rates strongly differed between proteins and were tested for their biological significance through statistical correlations with relevant parameters such as codon or amino acid bias. These efficiencies and degradation rates were not constant in all growth conditions and were inversely proportional to the growth rate, indicating a more efficient translation at low growth rate but an antagonistic higher rate of protein degradation. Estimated protein median half-lives ranged from 23 to 224 min, underlying the importance of protein degradation notably at low growth rates. The regulation of intracellular protein level was analysed through regulatory coefficient calculations, revealing a complex control depending on protein and growth conditions. The modeling approach enabled translational efficiencies and protein degradation rates to be estimated, two biological parameters extremely difficult to determine experimentally and generally lacking in bacteria. This method is generic and can now be extended to other environments and/or other micro-organisms.

## Author Summary

This work is in the field of systems biology. Via an in-depth comparison of proteomic and transcriptomic data in various culture conditions, our objective was to better understand the regulation of protein levels. We have demonstrated that bacteria exert a tight control on intracellular protein levels, through a multi-level regulation involving translation but also dilution due to growth and protein degradation. We have estimated translational efficiencies and protein degradation rates by modeling. These two biological parameters are extremely difficult to measure experimentally and have not been previously determined in bacteria. We have found that they are growth rate dependent, indicating a fine control of translation and degradation processes. We have worked with the small genome bacterium *Lactococcus lactis* on a limited number of mRNA-protein couples but keeping in mind that this approach could be extended to other micro-organisms and biological phenomena. We have exhibited that mathematical modeling associated to experimental steady-states cultures is a powerful tool to understand microbial physiology.

## Introduction

In the era of “omics”, systems biology has emerged with the availability of genome-wide data from different levels, *i.e.* genome, transcriptome, proteome, metabolome [1],[2]. This approach aims at integrating omics data, mainly through computational and mathematical models [3],[4] so as to decipher biological systems as a whole [5]. The integration of transcriptomic and proteomic results is a huge challenge by itself. The literature usually exploits these two approaches as complementary tools and does not often provide a correct confrontation of the two datasets. Until now, only a few researchers, mainly interested in yeast physiology [6],[7], have been working on this aspect and the results typically revealed modest correlations between those two datasets [8]–[10]. These weak correlations between transcript and protein levels can be the consequence of the involvement of post-transcriptional regulations [11], such as translation control and protein degradation as evidenced by Brockmann *et al.* [12]. Translation regulations are believed to be involved in protein level control but are generally studied at the level of controlling specific molecular mechanisms and not at the genome scale [13]–[15]. Although polysome profile analysis allows translation efficiencies to be experimentally determined for the various transcripts simultaneously, this technique has been only rarely used and almost exclusively for *S. cerevisiae* [16]. Protein stability can also influence intracellular protein level and the correlation between transcript and protein [10],[17],[18]. However protein stability is rarely studied at the genome scale and data are only available for *S. cerevisiae* [19],[20]. Finally, the rate of protein disappearance due to protein dilution by cellular growth is also potentially involved in protein level modifications but this physical phenomenon is generally neglected. More generally, even if translation efficiency, protein degradation and dilution rate can all influence protein levels, these parameters are not usually studied simultaneously. The role of each parameter in a whole cellular adaptation process has not been elucidated and it is not clearly known today which parameter is preponderant and if the control is constant or not when environmental conditions are modified.

The aim of this study was to analyse the control of intracellular protein level taking into account all the parameters of this control, in a prokaryotic organism, the model of lactic acid bacteria, *Lactococcus lactis*. To achieve this purpose, transcriptomic and proteomic analyses were performed with cells from the same culture. Transcriptomic data were already available [21] and the corresponding proteome measurement was performed. The whole protein related processes including translation, dilution rate and protein degradation were modelled, and, since biological data were obtained at steady state, equations describing the protein levels equilibrium were solved. This modeling approach allowed translation efficiency and protein degradation to be estimated and the relative involvement of all the various parameters of protein control to be analysed.

## Results

### Transcriptomic - Proteomic data

*L. lactis* was grown in continuous culture at different growth rates in the conditions previously described [21] and samples were taken for both transcriptome and proteome analysis at three dilution rates, *i.e.* 0.09, 0.24 and 0.47 h^−1^: the lowest growth rate (μ = 0.09 h^−1^) was chosen as reference. Despite the small size of the *L. lactis* genome (2310 genes [22]), a total of 346 different proteins were quantified corresponding to 308 different proteins measured in each repetition for each of the 3 steady states. Among these proteins, 193 showed differential profiles in response to a growth rate increase: 88 with reduced level and 105 with higher level. All the proteins displaying a significant level of modification for at least one of the dilution rates are listed in Table 1. In accordance with what has previously been found with transcriptome analysis [21], increased levels of proteins related to biogenesis were observed when the growth rate was increased, *i.e.* proteins related to transcription (GreA, NusA, QueA, RpoA), translation and more specifically ribosomal proteins (GatA, GatB, RplE, RplI, RplJ, RplK, RplM, RplN, RpmE, RpsA, RpsF, RpsT), enzymes related to fatty acid and phospholipid metabolism (AccA, AccD, FabD, FabF, FabG1, FabH, FabZ1, HmcM, ThiL, YdiD, YscE), two proteins involved in cell division (FtsY, FtsZ), and some proteins associated with purine, pyrimidine, nucleoside and nucleotide metabolism (Add, Adk, Apt, DeoB, GuaA, GuaC, Hpt, NrdE, PydA, PyrC, PyrE, RmlA, RmlB, Upp).

**Table 1:** List of proteins ordered by functional category and changing when growth rate increases from 0.09 to 0.24 and 0.47 h^−1^ during continuous culture of *L. lactis*.

	+	Proteins significantly over-expressed in response to growth rate increase	−	Proteins significantly under-expressed in response to growth rate increase
**FUNCTIONAL CATEGORY**	0.24 h^−1^/0.09 h^−1^	0.47 h^−1^/0.09 h^−1^	0.24 h^−1^/0.09 h^−1^	0.47 h^−1^/0.09 h^−1^
**AMINO ACID BIOSYNTHESIS**	AroH^1.60^, IlvA1^.66^, IlvD^1.61^, LeuA^1.92^, LeuD^2.20^, SerB^1.50^	AroE^1.16^, IlvD^1.43^, ThrC^1.13^	AspB^0.74^, GlnA^0.25^, LysA^0.48^, ProA^0.81^	AspC^0.57^
**BIOSYNTHESIS OF COFACTORS, PROSTHETIC GROUPS, AND CARRIERS**	CobQ^2.26^, IspB^1.82^	IspB^1.69^	DfpA^0.42^, GshR^0.59^	DfpA^0.44^
**CELL ENVELOPE**	MurC1^.42^, MurD^1.49^	MurC^1.68^	MurE^0.36^	
**CELLULAR PROCESSES**	DnaK^1.35^, FtsZ^1.83^, SodA^1.44^	FtsY^1.64^, GroEL^1.59^	AhpC^0.77^, SecA^0.70^	SecA^0.70^
**CENTRAL INTERMEDIARY METABOLISM**	GlmS^1.94^, MetK^1.23^	MetK^1.28^		GlgD^0.53^
**ENERGY METABOLISM**	ArcC2^1.51^, CitC^1.54^, DxsB^1.27^, Glk^1.99^, GpdA^1.38^, Mae^1.49^, NdrI^2.32^, Pyk^1.44^, TpiA^1.47^, YpjF^1.56^, YpjH^2.19^	CitE^1.20^, EnoB^1.45^, GpdA^1.41^, Mae^1.04^, Pmg^1.14^, TpiA^1.52^	AckA2^0.37^, ArcA^0.28^, CitF^0.60^, NifS^0.62^, PdhA^0.56^, PdhB^0.72^, PdhC^0.44^, Pfl^0.58^, RpiA^0.78^, YpdB^0.50^, YpdC^0.49^, YpdD^0.57^, YrcA^0.51^, YrjC^0.39^	AckA2^0.32^, AldC^0.73^, ArcT^0.65^, GadB^0.73^, GalE^0.98^, PdhA^0.78^, PdhB^0.71^, PdhC^0.38^, Pfl^0.93^, Pgk^0.85^, PycA^0.57^, ScrK^0.78^, YbiE^0.79^, YpdB^0.58^, YpdD^0.51^, YrbA^0.73^, YrcA^0.50^
**FATTY ACID AND PHOSPHOLIPID METABOLISM**	AccA^2.50^, AccD^1.82^, FabD^2.45^, FabH^1.47^, HmcM^1.61^, YdiD^2.07^	FabF^1.01^, FabG1^1.16^, FabH^1.54^, FabZ1^1.06^, ThiL^1.69^	PlsX^0.67^	YscE^0.86^
**PURINES, PYRIMIDINES, NUCLEOSIDES AND NUCLEOTIDES**	Adk^1.46^, Apt^2.40^, GuaC^2.43^, Hpt^1.34^, NrdE^1.57^, PyrC^1.31^, PyrE^1.77^, RmlA^1.71^, RmlB^1.14^	Add^1.49^, Apt^2.84^, GuaA^1.52^, Hpt^1.31^, PydA^1.18^, PyrC^1.42^, RmlB^1.29^	Pdp^0.80^, PurB^0.47^	DeoB^0.92^, PurB^0.38^, Upp^0.80^
**REGULATORY FUNCTIONS**	LlrC^1.33^, ObgL^1.44^, PurR^1.17^, PyrR^1.67^	ObgL^1.28^	CcpA^0.87^, EraL^0.57^, FhuR^0.75^	EraL^0.61^, FhuR^0.81^, LlrA^0.81^, YsxL^0.88^
**REPLICATION**	SsbB^1.64^		HslA^0.67^, ParC^0.34^	HslA^0.63^, ParC^0.34^
**TRANSCRIPTION**	GreA^1.17^, NusA^1.28^	QueA^1.31^, RpoA^1.08^	-	-
**TRANSLATION**	Frr^1.54^, LeuS^1.66^, PpiB^1.79^, PrfA1^.48^, RplE^1.38^, RplJ^2.81^, RplM^1.23^, RplN^1.34^, RpmE^1.07^, RpsA^1.20^, RpsF^1.45^, RpsT^1.19^, SerS^1.27^, TrpS^1.77^, Tsf^1.16^, Tuf^1.11^, TyrS^1.45^	FusA^1.37^, GatA^1.35^, GatB^1.35^, RplE^1.40^, RplK^1.07^, Tsf^1.26^	ArgS^0.55^, GltX^0.62^, PepP^0.19^, ProS^0.68^, RplA^0.73^, SerS^0.74^	ArgS^0.84^, KsgA^0.24^, LeuS^0.79^, LysS^0.42^, PrfC^0.63^, RplI^0.75^, RpsB^0.60^
**TRANSPORT AND BINDING PROTEINS**	GlnQ^1.20^, OptD^1.71^, PtsI^1.57^, PtsK^1.38^, YsfB^1.30^	GlnQ^1.19^, OptD^1.80^, PtsI^1.94^, YjgE^1.44^, YsfB^1.39^	BusAA^0.30^, PtsH^0.71^	BusAA^0.22^, PtsH^0.70^
**OTHER CATEGORIES**			ClpC^0.25^, CspE^0.62^, Pi102^0.74^, Pi125^0.47^	CspE^0.66^, DpsA^0.85^, Pi102^0.63^, Tpx^0.67^
**UNKNOWN**	YbdD^1.70^, YciC^1.22^, YcjB^1.60^, YejH^1.20^, YjgF^1.37^, YjhD^1.28^, YraB^1.88^, YshC^1.66^, YtfB^1.48^, YtjH^1.28^, YuhE^2.32^	YbdD^1.48^, YcjB^1.54^, YgbD^1.17^, YjgF^1.33^, YjhD^1.24^, YlaC^1.17^, YnhC^1.26^, YraB^1.79^	YahB^0.60^, YgdA^0.46^, YhjA^0.27^, YlaF0^.76^, YnfC^0.32^, YpdB^0.50^, YpdC^0.49^, YpdD^0.57^, YrjD^0.49^, YtgH^0.47^, YtjA^0.76^, YtjH^0.75^, YwcC^0.37^, YxbE^0.23^	YahB^0.61^, YcdB^0.58^, YeiJ^0.68^, YgdA^0.51^, YgiI^0.25^, YgiK^0.33^, YiiH^0.56^, YkhD^0.84^, YpdB^0.58^ YpdD^0.51^, YqfE^0.77^, YseF^0.93^, YtaA^0.81^

Protein expression ratios are indicated as exponent.

Proteome profiles differed between the various stress-related proteins. On one hand, the two chaperones DnaK and GroEL, the superoxide dismutase associated to oxygen stress SodA, and DpsA, were found in higher quantity, while on the other hand, the cold shock associated protein CspE, ClpC and the adaptation related peroxidase Tpx, had decreased levels in response to growth rate increase. Besides those opposite punctual regulations, other proteins encoding important functions involved in stress protection such as ATPases or peptidases (excepting PepP), were present at constant levels, independently of the growth rate. This lack of general tendency observed here at proteomic level was also observed at transcriptomic level [21]. In contrast, a wide down-regulation of genes involved in stress protection was observed in yeast when growth rate was increased [23],[24]. Finally, one can notice that the two single phage-related proteins measured in those proteomics experiments showed significantly reduced levels at high growth rate. This last observation can be connected with the previously described massive down regulation of the expression of phage-related genes [21].

**Figure 1:**
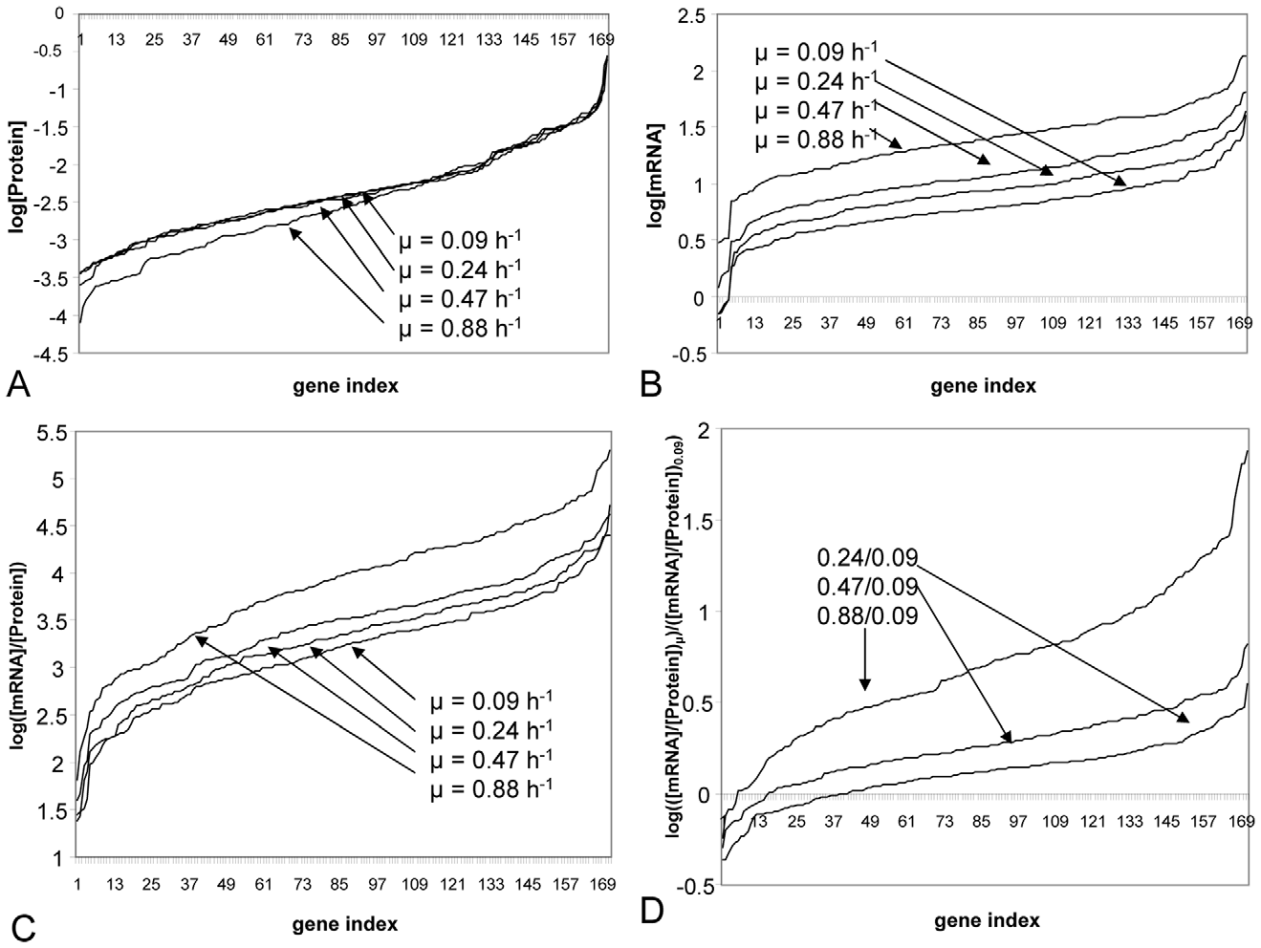
Distributions of protein and mRNA data for different growth rates in *L. lactis*. Protein concentrations (A), mRNA concentrations (B), mRNA/protein ratios (C) and their ratios between two different growth conditions (D) ranked in increasing order.

Transcriptomic and proteomic analyses were performed with cells collected simultaneously from the same fermentor; thus data can be strictly compared. Proteins and their corresponding transcript levels were compared individually. Transcriptomic data were already available [21] but were nevertheless re-processed so as to obtain concentrations rather than abundances (see Materials and methods). For proteomic data, concentration and abundance values are expected to be similar (see Materials and methods). For each growth rate, transcriptomic and proteomic mean values with their standard deviations are given in supplementary data (Table S1). The mRNA/protein ratios were not constant for the different genes since, at a given growth rate, data were spanned among five orders of magnitude (Figure 1C). These variations were linked both to protein and mRNA changes though protein concentrations were globally more spanned than mRNA concentrations (4 and 2 log of magnitude respectively; see figure 1A and 1B). mRNA/protein ratios were compared between two conditions using the lowest growth rate (0.09 h^−1^) as reference (Figure 1D) and they globally increased with the growth rate. This tendency was confirmed when we analyzed similarly data corresponding to the maximum growth rate of 0.88 h^−1^. These last data, also available in our group, were obtained in batch culture during the exponential growth phase, since this high growth rate could not be reached in continuous culture without any wash out of the cells from the chemostat [25].

### Modeling of cellular process

**Figure 2:**
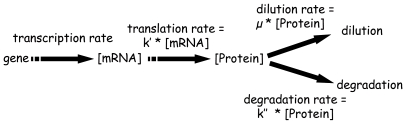
Modeling of the cellular process. Translation, dilution and degradation rates expressed respectively by k′[mRNA]), μ[protein] and k″[protein] where k′ is the translation efficiency, μ the growth rate and k″ the degradation rate constant.

What normally occurs in bacterial cells is the transcription of genes into mRNA, which are then translated into proteins that can be either diluted by growth or degraded (Figure 2). Hence, protein concentration is determined not only by translation rates but also by dilution and degradation, therefore the following balance equation can be written:
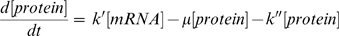
(1)


Rates of mRNA translation or protein degradation/dilution are assumed to be not constant and related to mRNA or protein concentration respectively. Biological rates were expressed as first order kinetics of their substrate concentration as previously postulated in *L. lactis* [26],[27] but also in yeast strains [19],[28]. Such modeling approach at the genomic scale is rare in the literature and dynamic experimental data allowing more elaborated kinetics to be hypothesized are not available. Making more complex those rate expressions would thus not make sense today. Dilution constant corresponds to the growth rate (μ), degradation constant (k″) is proportional to protein half-life (t_1/2_ = ln(2)/k″) and k′ represents the translation efficiency. At steady state, the various concentrations are expected to remain constant, the time derivative of the protein concentration is equal to zero and the previous equation can be simplified and reorganized as follows:
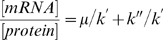
(2)


In the chemostat cultures at the various growth rates, cells are at steady state; similarly, during the exponential growth phase, cells are physiologically stable and are also considered to be at steady-state [29]. The previous observation, establishing a relationship between mRNA/protein ratios and the growth rate for these four steady states (see above), is in accordance with this last equation (2). 171 different mRNA and protein couples were available in each repetition of the various steady states (intersection of 308 couples in the 3 chemostat steady states and 191 in the batch). For only a few proteins were probes missing on the microarray; hence it was not possible to rebuild these couples. In order to estimate translation efficiency and protein degradation rate, the best mathematical solution to the equation (2) was sought, using numerical estimations performed on Matlab. The k′ and k″ values were postulated to be positive, in accordance with biological reality. Different solutions with k′ and/or k″ constant, directly or inversely proportional to μ were investigated. Estimation of the best fitting solution was based on the least square criterion [30]. For the 171 couples, the mean sum of the squared residuals (difference between a ratio and its estimation) associated to every combination are given in Table 2. Considering the lower mean sum of the squared residuals, the best solution was obtained when both k′ and k″ were proportional to 1/μ (k′ = α/μ and k″ = β/μ). Hence the equation (2) could be written as follows:
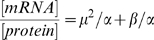
(3)


The mRNA/protein ratios were thus linked to the growth rate (μ) through a polynomial function of order two (μ^2^), which is consistent with the visual observation of the various curves (not shown). For each mRNA–protein couple the reliability of the two estimated constants α and β was evaluated by their associated R^2^. All regression coefficients are listed in Table 3. The mean linear coefficient (R^2^) associated to this model was 0.83±0.04. Finally, the consistency of our modeling approach was checked when removing the data of the batch exponential growth phase from the analysis. A high mean R^2^ of 0.77±0.12 was still obtained using chemostat data exclusively. On the contrary, data not at steady state coming from other growth phases in batch cultivation could not be included. Indeed when taking into account mRNA/protein values during growth deceleration or during stationary phase, R^2^ was strongly affected and dropped to 0.21±0.03 and 0.24±0.04 respectively.

**Table 2:** Mean sum of squared residuals associated to different solutions to solve equation (2).

	k″ = β	k″ = β*μ	k″ = β/μ
k′ = α	1.80E+08+/−1.21E+08	2.06E+08+/−1.24E+08	1.79E+08+/−1.21E+08
	*8.85E+05+/−5.21E+05*	*2.00E+06+/−1.03E+06*	*1.48E+06+/−1.25E+06*
k′ = α*μ	7.13E+08+/−4.21E+08	7.14E+08+/−4.21E+08	7.12E+08+/−4.21E+08
	*2.68E+06+/− 1.67E+06*	*2.20E+06+/−1.27E+06*	*2.71E+06+/−1.69E+06*
k′ = α/μ	7.80E+07+/−4.15E+07	1.13E+08+/−5.28E+07	**4.80E+07+/−2.97E+07**
	*1.79E+06+/−1.00E+06*	*2.62E+06+/−1.42E+06*	***5.69E+05+/−4.48E+05***

Transcriptomic data were expressed as mRNA concentrations and abundances (data in italic). The lowest mean sum of squared residuals, revealing the solution that best fits, is indicated in bold.

**Table 3:** Modeling of the mRNA/protein ratios by a linear relation of μ^2^/α+ β/α.

Protein and Functional category	α	β	R^2^
**AMINO-ACID BIOSYNTHESIS**			
AROH	4.51151E-05	0.1646825	0.97121488
GLTD	1.85762E-05	0.00241775	0.97233168
GLYA	4.22229E-05	0.01354879	0.93404229
*HOM*	*1.68474E-05*	*0.28617974*	*0.86672614*
ILVD	7.93403E-05	0	0.93524863
*LYSA*	*1/α = 0*	*1/α = 0*	*0*
THRC	0.000284821	0.29820117	0.98839848
**CELLULAR PROCESSES**			
AHPC	5.10745E-05	0	0.97664571
DNAK	0.001061111	0.35860785	0.93348064
FTSA	4.56985E-05	0.05202765	0.9606819
FTSZ	5.8834E-05	0.00130416	0.92637554
SECA	2.17858E-05	0	0.97642785
SODA	0.0001363	0.1338211	0.97950777
TIG	0.001348633	0.2948733	0.98174986
**BIOSYNTHESIS OF COFACTORS, PROSTHETIC GROUPS, AND CARRIERS**			
MENB	2.73156E-05	0	0.93563899
NADE	0.000116486	0.15449175	0.99604123
TRXB1	8.19857E-05	0	0.96026851
**CELL ENVELOPE**			
DDL	8.25457E-05	0.11519472	0.96225747
GLMU	7.37934E-05	0.09190161	0.99637455
*MURC*	*0.001375775*	*2.67713991*	*0.42558387*
*MURD*	*0.000185444*	*2.21919112*	*0.31988696*
MURF	2.2806E-05	0	0.93893948
**FATTY ACID AND PHOSPHOLIPID METABOLISM**			
ACCC	2.58433E-05	0.00441038	0.95136582
FABF	0.000184132	0.11832197	0.96441487
FABG1	0.007749352	19.3686308	0.00359184
FABZ1	0.00099817	1.03586823	0.8424436
HMCM	4.85365E-05	0.1262591	0.91591656
LPLL	6.97982E-05	0.32322579	0.90681314
THIL	4.11396E-06	0	0.92850481
**CENTRAL INTERMEDIARY METABOLISM**			
GLMS	0.000107736	0.58029666	0.70310455
METK	3.24175E-05	0.08257955	0.99050511
**ENERGY METABOLISM**			
ACKA1	7.48258E-05	0.10443145	0.98553347
ACKA2	3.87311E-05	0.00463101	0.96562819
ALS	3.63408E-05	0.1298218	0.99148164
ARAT	1.02213E-05	0.17625044	0.98935052
BCAT	0.000106471	0.19579547	0.98752533
CITE	0.000631153	1.91842211	0.33567603
CITF	0.000141916	0.21345432	0.89438864
DXSB	2.22939E-05	0.01305167	0.96347158
ENOA	0.001448014	0.50011755	0.9812687
FBAA	0.001567707	0.58952655	0.92631253
GALE	1.51579E-05	0.08891695	0.96369781
GAPA	6.96015E-05	0.01558052	0.98304437
GAPB	0.005918802	0.34027767	0.95321111
*GLK*	*9.40795E-05*	*3.25489872*	*0.07096222*
GPDA	2.89337E-05	0.01358186	0.95337614
LDH	0.001985093	0.19145278	0.99659282
MAE	0.000203098	0.11729054	0.94812409
NIFS	0.000102282	0.2123659	0.94633073
PDHA	0.000208532	0.07344591	0.99091991
PDHB	4.80055E-05	0.16285919	0.99397297
PDHD	0.0001525	0.10948091	0.99214624
PFL	6.25985E-05	0.08619004	0.99383945
PGK	0.000724945	0.18072841	0.99732793
PMG	0.00120216	0.52424582	0.95549172
*PYCA*	*0.000229023*	*0.62375415*	*0.67646518*
PYK	0.001608755	0.27146917	0.99772835
TKT	0.000162636	0.24758748	0.98112816
TPIA	0.001187986	0.562022	0.99194516
YPDB	0.000110296	0.17219254	0.93840544
YPDD	0.000120767	0.06394114	0.99533841
YPJH	2.02435E-05	0	0.90640904
*YRBA*	*3.46086E-05*	*0.40668707*	*0.87944016*
YRCA	4.99377E-05	0.2788185	0.95535145
ZWF	0.000467917	0.33052248	0.99925746
**OTHER CATEGORIES**			
CLPB	4.1254E-05	0.11738861	0.99444661
CLPE	1.97662E-05	0	0.95854444
*CSPE*	*0.000210856*	*0.88526205*	*0.54952575*
DPSA	0.000339167	0.14740893	0.99853144
**PURINES, PYRIMIDINES, NUCLEOSIDES AND NUCLEOTIDES**			
*ADK*	*0.000134926*	*0.62963333*	*0.86713456*
*DEOD*	*0.000465012*	*1.68108849*	*0.52943563*
GUAA	0.000155463	0.23586297	0.96859337
HPT	0.000108095	0.49984413	0.99936199
PDP	2.47401E-05	0.02445447	0.96991137
PRSB	0.000112449	0.15467248	0.91947476
*PURB*	*0.000804327*	*1.72339847*	*0.24260508*
*PYRB*	*1/α = 0*	*1/α = 0*	*0*
PYRC	8.87977E-05	0.28528728	0.93021038
*PYRE*	*1/α = 0*	*1/α = 0*	*0*
PYRH	6.38656E-06	0.0322555	0.95142351
RMLA	6.81773E-05	0.32316896	0.93695889
*RMLB*	*0.001325841*	*0.99636334*	*0.88101663*
RMLC	1.28376E-05	0.03849101	0.98656712
THYA	5.59309E-06	0	0.95171946
**REGULATORY FUNCTIONS**			
CCPA	0.000406211	0.12171163	0.96201171
CODY	8.46992E-05	0.13250482	0.98384633
LLRA	4.62605E-05	0.52331337	0.92153533
*LLRC*	*0.013424621*	*15.5080356*	*0.10463244*
PURR	0.000103485	0.03276367	0.94032578
*PYRR*	*0.028302603*	*16.3995592*	*0.00734326*
TYPA	0.000229966	0.69467632	0.90710663
**REPLICATION**			
DNAN	0.00066181	0.27185813	0.99667727
HSLA	0.003874397	0.14399314	0.98785442
*PCRA*	*7.04126E-05*	*0.47756787*	*0.69922849*
RECA	0.000347948	0.29995273	0.97437563
SSBB	4.73141E-05	0.06933065	0.96506692
**TRANSLATION**			
*ARGS*	*0.001086633*	*1.61719244*	*0.08263897*
ASPS	0.000293319	0.78275625	0.91713371
DEF	1.14893E-05	0.03005374	0.97404273
FMT	3.34181E-05	0	0.94159277
*FRR*	*0.001064713*	*2.12697168*	*0.32948552*
*FUSA*	*0.017698731*	*19.1276933*	*0.01754756*
GATB	0.000154178	0.16452557	0.97872462
*LEUS*	*0.000196012*	*0.87373537*	*0.39629741*
*LYSS*	*0.000190963*	*1.59349084*	*0.12786788*
*METS*	*4.66627E-05*	*0.33966148*	*0.83515216*
PEPC	2.36902E-05	0	0.92969273
PEPDB	0.000204376	0.2243337	0.99552578
*PEPN*	*1/α = 0*	*1/α = 0*	*0*
PEPO	0.000147934	0.15508792	0.99642862
PEPP	1.72239E-05	0	0.91413837
*PEPT*	*1/α = 0*	*1/α = 0*	*0*
PEPV	0.00071641	0.29661492	0.99715732
PHET	0.000187455	0.76933426	0.98165509
PPIB	4.99542E-05	0.24407188	0.97980974
*PRFA*	*3.0659E-05*	*0.58910602*	*0.85034465*
PRFC	5.60905E-05	0.30692988	0.95144129
PROS	8.83818E-05	0.11998473	0.99431058
RPLA	0.001100954	0.29255803	0.93892795
*RPLC*	*0.002415577*	*0.86045223*	*0.31939893*
RPLF	0.020868509	0.57572422	0.92991859
RPLI	0.00053669	0	0.96573657
*RPLJ*	*0.006454043*	*1.31313441*	*0.34869518*
RPLK	0.007187335	0.15914489	0.98448783
RPLN	0.002583908	0.42853325	0.9592895
RPLQ	0.000971518	0.16634258	0.99841319
*RPME*	*0.000391868*	*0.73950729*	*0.77695301*
*RPSA*	*0.001602607*	*0.73935178*	*0.85488589*
*RPSB*	*0.00107443*	*3.19530018*	*0.01500431*
RPSC	0.000377121	0	0.95023628
RPSD	0.000400639	0.00974974	0.99060528
*RPSE*	*0.001769065*	*0.47234255*	*0.8144781*
RPSF	9.14703E-05	0.48098276	0.9965725
*RPSG*	*0.004263125*	*0.78803197*	*0.87015105*
RPSH	0.000342902	0.17581565	0.98687861
RPSJ	0.000193424	0	0.95720261
RPST	0.001476284	0.3612539	0.97646346
*SERS*	*0.088336502*	*91.1163917*	*0.0004214*
TRPS	2.10306E-05	0	0.95229578
TSF	0.00086506	0.31609137	0.97370399
TYRS	0.001325453	0.49617209	0.94131316
**TRANSCRIPTION**			
GREA	0.000177242	0.18909676	0.94390233
NUSA	0.000550647	0.23668226	0.97440049
*QUEA*	*0.000373228*	*6.34517377*	*0.0865523*
RPOA	3.42628E-05	0.13144055	0.98711891
**TRANSPORT AND BINDING PROTEINS**			
PTNAB	0.000173226	0.13096684	0.97180887
*PTSH*	*1/α = 0*	*1/α = 0*	*0*
PTSI	0.000457181	0.23653753	0.90691365
PTSK	4.97924E-05	0.00417517	0.95466442
YAHG	6.35546E-05	0.32396408	0.94254081
YNGE	4.06269E-05	0.02850911	0.955139
YSFB	4.93442E-05	0.14214495	0.96437898
**UNKNOWN**			
YAHB	3.18423E-05	0	0.97419179
YBJJ	1.86559E-05	0	0.92494911
YCGE	9.62931E-05	0.24684603	0.98565885
YCIC	0.000121264	0.33790758	0.97435427
YDJD	2.16629E-05	0.04413265	0.95721016
YEIG	5.2701E-06	0	0.92020873
YNIH	5.57972E-05	0.24822788	0.93523297
YPDC	2.62297E-05	0.05529531	0.97485946
YRAB	0.000214255	0.05292201	0.94877672
YSEF	1.45972E-05	0	0.97271095
YTAA	1.42892E-05	0	0.92865164
YTDB	4.44976E-05	0.12699478	0.99961866
YTGG	7.14082E-06	0.07061493	0.99826851
YTGH	1.26964E-05	0	0.92451445
YTHC	6.60287E-05	0	0.95043811
YTJH	4.18078E-05	0.23305714	0.98316698
YUHE	1.12397E-05	0	0.91482774
YWCC	0.000120121	0.04546948	0.9672213
YWED	1.52202E-05	0	0.92566783

α and β estimations and the determination coefficient (R^2^) are given for the 171 genes for which both transcriptomic and proteomic data were available. α and β are directly proportional to translation and degradation rates respectively (k′ = α/μ and k″ = β/μ). Proteins that do not match the selection criteria (R^2^≥0.90) are *italicized.*

The model (1) states that translation rate is proportional to the concentration of mRNA species which assumes that translation is mRNA-limited. An alternative hypothesis, would be the saturation of the ribosome with mRNA, as previously postulated in *E coli* [31], which implicitly suggests competition of any specific mRNA with all others to be the determining factor in synthesis of the corresponding protein. We have thus tested the model (2) with mRNA abundances rather than concentration values. The modeling approach was robust since similar k′ and k″ dependencies to the growth rate (k′ = α/μ and k″ = β/μ) were obtained (Table 2; data in italic). However the expression of individual protein/mRNA as a function of μ^2^ (3) had generally lower R^2^ (only 48 couples with R^2^≥0.90 compared to 130 with concentrations), indicating the modeling approach with abundances was less satisfactory than with concentrations.

### Model predictions and biological relevance

In order to carry on our modeling approach, the data corresponding to mRNA concentrations were filtered and only couples with R^2^≥0.90 (130) were retained for further analyses (Table 3). It could be noticed that among the 41 eliminated couples, 15 displayed non monotonous evolutions of their mRNA/protein ratios against μ^2^ and 20 had very low mRNA/protein ratios, which were thus more sensitive to errors.

**Figure 3:**
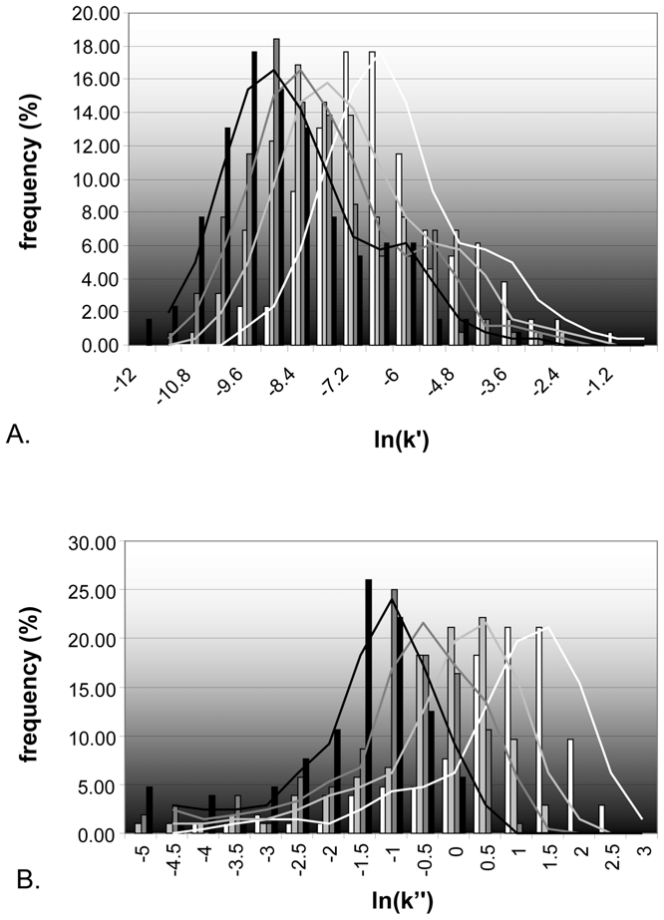
Distribution of translation efficiency and degradation rate constant for different growth rates in *L. lactis*. Histograms for translation efficiency, k′, (A) and protein degradation rate, k″, (B) with coloured bars (black for μ = 0.88 h^−1^, dark grey for μ = 0.47 h^−1^, grey for μ = 0.24 h^−1^ and white for μ = 0.09 h^−1^) and lines indicating the Gaussian tendency curves.

The k′ and k″ coefficients were numerically calculated for each protein and in each growth condition from the values of α and β (Table 2) by the relation k′ = α/μ and k″ = β/μ. Value distributions (Figure 3) demonstrate the wide variability of k′ and k″ among proteins but also between growth conditions. The k″ decreases when growth rate increases which is consistent with the general idea that protein degradation is high in stationary phases [32]. Protein half-lives were calculated and median values of t_1/2_ were respectively 23, 61, 119 and 224 min for 0.09, 0.24, 0.47 and 0.88 h^−1^. These values are in the same order of magnitude as those obtained recently for *S. cerevisiae* (mean 43 min at a growth rate of 0.1 h^−1^ [19]). Like k″, the translation efficiency k′ is also expressed as 1/μ function, indicating that, when the degradation process increases at low growth rate, the translation efficiency is also increasing in order to attenuate this negative biological effect.

Due to the restricted size of the dataset but also to the non-uniform distribution of detected proteins in the various functional categories, it was not possible to use statistical tests to rigorously determine functional enrichments in extreme values of k′ or k″. However, among the 15 genes that are translated the most efficiently (highest α values in Table 3), one can notice the over-representation of genes involved in major cellular processes: the Tig chaperone [33] and proteins involved in replication (HslA, which can unwind DNA and plays a role in its supercoiling, [34]), and translation (ribosomal proteins: RplA, RplF, RplK, RplN, RpsT). Carbon metabolism is also represented by 7 proteins (GapB, EnoA, FbaA, Pmg, Pyk, TpiA, Ldh), all belonging to glycolysis which is the major metabolic pathway for energy production in *L. lactis*. The extremely stable proteins correspond to null values of β, and consequently k″, were represented by a group of 26 proteins. Remarkably, half of them were related to stress responses: ClpE protease, PepC and PepP peptidases, three reductases that are usually linked to oxidative stress (AhpC, TrxB1, YpjH), but also MurF, involved in parietal structure, YtgH, which is homolog to *Staphylococcus aureus* alkaline stress protein [35], YtaA and YahB two hypothetical protein sharing homologies with *E. coli* universal stress protein Usp [36], YuhE, whose *E. coli* homologue is involved in copper resistance [37], and two cysteine desulfurases (YeiG and YseF) whose corresponding genes in *E. coli* are involved in oxygen and copper stress responses [38]. Moreover, those extremely stable proteins are rather in the last third for translation efficiency. Thus *L. lactis* may limit degradation of stress-related proteins so as to maintain a minimal pool ready to use in case of emergency, which is biologically relevant.

Biological determinants of translation efficiency and protein stability were investigated through correlation studies. Correlations providing a Spearman coefficient (R_Spearman_) with associated p-values lower than 0.05 were considered as significant. The codon adaptation index (CAI) positively correlates with k′ (R_Spearman_ = 0.57). Since CAI directly reflects translation efficiency during the elongation step [39], this result validates our translation efficiency estimations. Translation efficiency is also tightly related to the amino acid composition of proteins. A negative correlation of k′ was obtained with tyrosine, cysteine, histidine, aspartic acid and isoleucine frequencies while lysine and alanine richness had a positive influence (Table 4). The amino acids the most used have a positive influence on k′ whereas those with a negative effect are the less frequent ones (Table 4). The single exception is for isoleucine, but since it is the limiting nutrient it is not surprising to find it negatively correlated with translation efficiency, despite its high frequency in *L. lactis* proteins. This amino acid bias, together with the codon bias (CAI), shows that translation efficiency is strongly dependent of the gene sequence. This optimized functioning state is probably the result of a long evolutionary process. Finally it was found that translation efficiency is affected by protein length: the longer the protein, the more k′ decreases (R_Pearson_ of −0.18). This negative correlation with length has already been reported for yeast [19] and can possibly be explained by a decrease of the ribosome density on long mRNA as previously shown for *S. cerevisiae* [40]. The only apparent correlation emerging for protein degradation constants k″ is a negative influence of cysteine richness (Table 4).

**Table 4:** Correlation analysis between amino-acid usage in *L. lactis* proteins and translation efficiencies or degradation rates.

Amino-acid	Mean usage frequency (%/protein)	Correlation with translation efficiency (R_Spearman_)	Correlation with degradation rate (R_Spearman_)
Cysteine	0.5	−0.29	−0.19
Tryptophan	1.1		
Histidine	1.8	−0.22	
Methionine	2.6		
Proline	3.0		
Tyrosine	3.6	−0.21	
Glutamine	3.6		
Arginine	3.7		
Phenylalanine	4.8		
Aspartic acid	5.1	−0.17	
Asparagine	5.1		
Threonine	5.5		
Glycine	6.2		
Serine	6.4		
Valine	6.5		
Alanine	7.0	0.23	
Glutamic acid	7.1		
Isoleucine	7.9	−0.19	
Lysine	7.9	0.24	
Leucine	10		

These correlations were independent of the growth rate. Significant R_Spearman_ with p-value<0.05 are listed in the table.

### Controlling mechanisms

**Figure 4:**
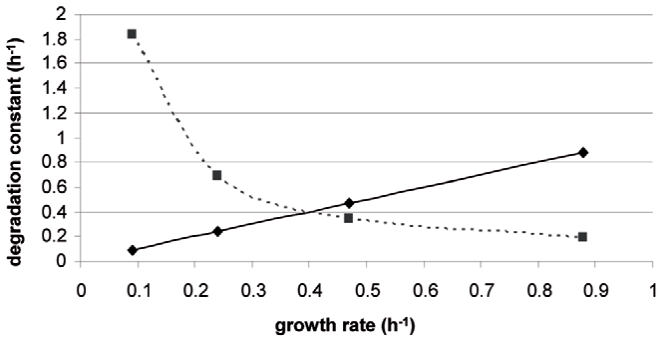
Competition between dilution and degradation rates for protein control. Comparison of growth rate (μ: straight line) and median value of the degradation constant (k″: dotted line).

Degradation and dilution by growth are both involved in protein disappearance and are competitive reactions. The degradation and dilution constants, k″ and μ, can be directly compared. The k″ is higher than μ at low growth rate but becomes lower after a critical value of 0.39 h^−1^ (Figure 4). The role of the degradation may thus be major at low growth rate while dilution may become the main phenomenon at fast growth.

More generally, variations in protein concentration between two conditions can be related to changes in the three rates: protein synthesis, degradation and dilution. In order to better understand this regulatory node and identify which are the major controls, the quantitative involvement of the different actors was analyzed. Regulation coefficients corresponding to the protein level control were estimated with a method based on the one developed on *S. cerevisiae* [41],[42]. Derivation of equation (2) leads to the following relationship:
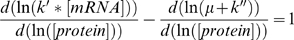
(4)


The term 
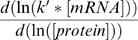
 of the equation (4) represents translation control on protein concentration and is called ρ_t_ while the term −
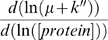
, named ρ_d_, includes both the dilution and the degradation and represents protein control by disappearance. ρ_t_ and ρ_d_ were estimated for each growth rate interval (between 0.09 and 0.24 h^−1^; between 0.24 and 0.47 h^−1^; between 0.47 and 0.88 h^−1^). The values of ρ_t_ were used to elucidate the nature of the control and are given in supplementary data (Table S2). If ρ_t_≤0, protein disappearance is the major controlling mechanism; if ρ_t_≥·1, it is translation; and if 0·< ρ_t_·< ·1, the control of protein is shared. The nature of the control for a given protein and its strength differed in the various the growth rate intervals. However a constant control by disappearance was observed for 6 proteins distributed all over the metabolism (Als, GreA, LplL, PyrC RplQ, ThrC, see Table 3 for associated functions). Inversely the unknown protein YpdC was the single one constantly controlled by translation process. Independently of the growth rate, protein levels are mostly controlled by disappearance (Table 5). Translation control strongly decreases, and protein level control becomes less specific and more and more shared with increasing growth rates. Similar conclusions were valid when ρ_d_ was used for the control analysis instead of ρ_t_ (data not shown).

**Table 5:** Protein control analysis in the different ranges of growth rate based on ρ_t_ calculation.

Growth rates intervals	Translation control of protein levels	Disappearance control of protein levels	Shared control of protein level
0.09–0.24 h^−1^	38%	60%	2%
0.24–0.47 h^−1^	33%	56%	11%
0.47–0.88 h^−1^	14%	47%	39%

## Discussion

The comparison of mRNA and protein ratios revealed a strong heterogeneity among genes but also for a given gene, at different growth conditions. Variability among genes has recently been reported for the model yeast *S. cerevisiae* but these ratios remained constant between the two studied conditions, *i.e.* a rich and a poor media [43]. Though lacking in this publication, the maximum growth rates of *S. cerevisiae* were estimated to be 0.46 and 0.35 h^−1^ respectively in a rich and a poor media (Parrou J.L., personal communication). Thus it is postulated that the growth rate difference between these two conditions was too small to induce changes in mRNA/protein ratios.

The combination of two modeling approaches, one based on biological knowledge and the other on experimental data fitting, has enabled translation efficiency and protein degradation rate to be determined for each protein, phenomena which have been shown to be protein specific and growth rate dependant. The positive correlation of translation efficiency with codon bias in *L. lactis* is consistent with the results obtained for the yeast, though translation efficiencies have been calculated differently [12]. The presence of genes related to major cellular processes essential for growth were marked among the best translated. This finding corroborates what was found in archaebacteria for ribosomal proteins [44]. In *L. lactis*, the growth-rate dependant variations in translation efficiency are probably not related to changes in the amount of intracellular ribosomes if the constant ratio between mRNA and ribosomal RNA (see Material and methods) is taken into account. However one has to bear in mind that rRNA does not necessarily means assembled and/or active ribosomes. It is known for example that *E. coli* ribosome activity can be modulated by the inter-conversion between a functional 70S and a dimerized 100S inactive form [45]. To resolve this question, it will be necessary to investigate genome-wide ribosomal activity via polysome distribution which would provide key information to decipher the regulatory processes controlling translation. Polysome profile technology is already available for yeast but may be difficult to adapt to bacteria due to the co-localisation of transcription and translation in the cytoplasm.

Protein half-lives for the whole genome have never been determined nor estimated in any bacteria and data are only available in the literature for *S. cerevisiae*. However studies disagreed in terms of average half-life values: 31 h for Pratt *et al.* against 43 min for Belle *et al.* [19],[20]. Those differences could be explained by methodological reasons since one study used pulse chase experiments [20] whereas the other one consisted in a direct measurement of each epitope-tagged proteins [19]. In our study, for *L. lactis*, protein median half-lives ranged from 23 to 224 min. These low values are in good agreement with most recent values obtained for *S. cerevisiae* [19] and indicate that protein degradation is considerably more rapid than was once believed. Degradation rates in *L. lactis* were negatively correlated to cysteine content in proteins. In yeast, stable proteins were previously found to have a higher valine density whereas unstable ones are enriched in serine [19]. It is difficult to strictly compare those results since amino acid bias may be species specific and reflect the particularities of proteases involved in protein degradation. The negative correlation with cysteine could nevertheless be related to the potential formation of disulfide bridges known for stabilizing proteins [46]. The current work also revealed the presence of stress related proteins among the most stable. This last observation differs from results obtained in yeast indicating that ribosomal proteins and enzymes from amino-acids metabolism have the higher half-life [19]. This high stability of stress protein together with the lack of global transcriptional stress response observed in *L. lactis* when the growth rate is changed clearly underlines differences of stress adaptation mechanisms between the two micro-organisms.

Protein degradation exerts a major role in the cellular adaptation process since protein half-live data depend on the growth rate (1/μ function). Moreover, the degradation rate is even higher than dilution rate at low growth rate (Figure 4). Considering that protein degradation is an ATP consuming process [47], high protein degradation at slow growth rate may contribute to the increase of maintenance energy that is generally observed in such conditions [48]. Like protein degradation, translation efficiency is also increased at slower growth rates. Effects of translation efficiency and protein degradation are thus antagonist and this mode of regulation is probably dedicated to attenuate biological changes. Inversely, proteins with the lowest degradation rates also corresponded to low translation efficiencies. The analysis of the regulation involved in the control of protein concentrations demonstrated that it is not constant in the different ranges of growth rate. At low growth rates, disappearance seems to be the main controlling mechanism, which could be attributed to high degradation rate. At high growth rate, the control becomes more complicated with some proteins regulated at the level of synthesis, disappearance or both (shared control). This increased complexity is consistent with cells approaching their maximum growth performance.

With this modeling approach, we have estimated translational efficiencies and protein degradation rates. These two biological parameters are extremely difficult to measure experimentally and have even never been previously determined in bacteria. The method was based on an in depth comparison of proteome and transcriptome data and was developed with the small genome bacterium *L. lactis* on a limited number of mRNA - protein couples (171). It will be possible in the future to broaden these couples since other proteomic methodologies, such as the APEX technology [8], allow more proteins to be detected. The approach remains generic and can be applied to all microorganisms. Modelling equations were solved because steady-states cultures were used: chemostat fermentation technology enabling steady states to be studied has thus proved to be a powerful tool to understand microbial physiology. We have demonstrated that bacteria exert a sharp control on intracellular protein levels, through a multi-level regulation involving three growth rate dependant actors: translation, dilution and degradation. Here, the growth rate was changed *via* chemostat cultures, but such growth rate modifications are also encountered in nature when cells have to face new environments. In this case, the adaptation process involves growth rate adaptation as well as other specific metabolic adaptations. It remains to be determined how the protein control is exerted in such natural environment.

## Materials and Methods

### Growth conditions

*Lactococcus lactis* ssp. *lactis* IL1403, whose genome has been entirely sequenced [22], was grown as previously described [21]. Briefly, three different growth rates have been studied, namely 0.09, 0.24 and 0.47 h^−1^ during anaerobic chemostat cultures (under nitrogen atmosphere and regulated pH) on a chemically defined medium limited by isoleucine concentration. For each steady-state, samples have been harvested in at least quadruplicate with a minimum delay of five doubling time between each sampling.

### Transcriptomic data reprocessing

Transcriptomic data (geo platforms GSE10256 [21] for chemostat culture and GSE12962 for batch exponential phase) were already available. Briefly, these transcriptomic analyses had been obtained with a constant amount (10 µg) of total RNA (mRNA, ribosomal RNA and transfer RNA) labeled by retro-transcription (^33^P) and hybridized on nylon membrane as previously described [49]. Three independent biological repetitions were used. These transcriptomic data had been normalized by all spots' mean intensity and thus corresponded to mRNA abundances. They were reprocessed here in order to calculate mRNA concentrations with the method previously described [49]. Raw data were first standardized by the all spots' mean intensities of the reference membrane (and not with its proper membrane) in order to eliminate the bias of the radioactivity level between the various repetitions and then corrected by total RNA concentration in order to take into account changes in intracellular RNA yield in the cells. Consistent with previous results [50], this yield increased significantly with the growth rate in *L. lactis* (3.58±0.39, 4.92±0.52, 7.34±0.28 and 11.06±0.23 g for 100 g cell dry weight at μ = 0.09, 0.24, 0.47 and 0.88 h^−1^ respectively). Since the amount of RNA to perform transcriptomic analysis is maintained constant in order to avoid retro-transcription labelling bias, these RNA yield changes are completely hidden by the technology.

The total raw intensity of the membrane without any normalisation represents the amount of mRNA in the RNA sample used for transcriptomic analysis (10 µg). This total intensity was constant at each growth rate and lower than the saturation threshold (mean value of 1660±584, 1462±383, 1389±366, 1474±367, 1496±425 at μ = 0.09, 0.24, 0.47 and 0.88 h^−1^ respectively). Thus, it can be deduced that the ratio mRNA/total RNA was constant and assuming that ribosomal RNA is the major component of total RNA we can postulate that the fraction mRNA/ribosomal RNA is independent of the growth rate.

### Proteomic analyses

For each condition, three repetitions were performed with independent cultures, extractions and electrophoresis. Bacteria were harvested from the cultures and cell pellets were washed twice with ice-cold 200 mM Na-phosphate, pH 6.4 and re-suspended in 4 ml of 20 mM Na-phosphate buffer, pH 6.4, 1 mM EDTA, 10 mM tributylphosphine, a cocktail of protease inhibitors (P8465; Sigma Aldrich, St Louis, MO) 20-fold diluted and catalase 40 U/ml (C3155; Sigma Aldrich, St Louis, MO) to limit isoform formation. The cell suspension (approximately 35 units of optical density at 600 nm [OD600]/mL,) was transferred to the pre-cooled chamber of a BASIC Z cell disrupter (Celld, Warwickshire, United Kingdom) and was subjected to a pressure of 2,500 bars. The suspension was centrifuged at 5,000×*g* for 20 min at 4°C to remove unbroken cells and large cellular debris. The supernatant was collected and centrifuged at 220,000×*g* for 30 min at 4°C. The total protein concentration in the resulting supernatant (cytosolic fraction) was determined with the Coomassie protein assay reagent (Pierce, Rockford, IL) using bovine serum albumin as standard and was included between 1 and 2 mg/mL. The cytosolic fraction was aliquoted and stored frozen at −20°C.

#### 2-Dimensional electrophoresis

A volume of cytosolic fraction corresponding to 350 µg or 500 µg (for basic gels) of proteins was incubated with nuclease (benzonase, Novagen 70664-3; 25 U for 100 µL of cytosolic fraction) for 30 min at 37°C and then chilled on ice and precipitated with 75% (vol/vol) methanol. The protein pellet was resuspended in 500 µL (for pH 4.5–5.5 and 5–6 gels) or 100 µL (for pH 6–11 gels) of isoelectric focusing (IEF) buffer 1, consisting of 7 M urea, 2 M thiourea, 4% CHAPS{}, 100 mM dithiothreitol or 4 mM tributylphosphine and DeStreak (1.2% v/v, Amersham Biosciences, GE Healthcare) (for basic gels), and 0.5% pH 4.5 to 5.5 or 5 to 6 or 6 to 11 immobilized pH gradient (IPG) buffer (Amersham Biosciences, GE Healthcare). The sample was loaded on 24 cm pH 4.5 to 5.5 or 5 to 6 IPG strip (Amersham Biosciences, GE Healthcare) which was previously rehydrated at 50 V for 11 h. IEF was carried out for 65,000 V.h at a maximum of 8,000 V, using the Protean II IEF cell (Bio-Rad, Hercules, CA). Analysis of basic proteins was performed with 18 cm pH 6–11 IPG strip. After passive rehydration of the strip in buffer 1, the protein sample was loaded on sample cups and IEF was carried out for 20,000 V.h at a maximum of 3,500 V using the IPGphor device (Amersham Biosciences, GE Healthcare). Before the second dimension, IPG strips were incubated for 15 min with shaking in 150 mM Tris-HCl pH 8.8, 0.1% w/v SDS. The IPG strip was then positioned on sodium dodecyl sulfate-polyacrylamide gels, using 1% low-melting-point agarose in 150 mM Tris-HCl, pH 8.8. Second-dimension electrophoresis was performed on 12% polyacrylamide gels (24 by 20 by 0.1 cm) in 25 mM Tris, 192 mM glycine, 0.1% sodium dodecyl sulfate, pH 8.3, using the Ettan-Dalt II apparatus. Electrophoresis was run at 1 W/gel for 16 h at 15°C. The gels were stained with BioSafe colloidal Coomassie blue (Bio-Rad) for 1 h and destained with three successive washes in deionized water.

Images files were recorded at 65536 gray levels (16 BitsPerPixel). Image manipulation and analysis were performed with Samespot V2 software (Nonlinear Dynamics). Protein abundances were given using arbitrary units which correspond to spot volumes and which were calculated as follows: spot area x spot pixel intensity - background intensity.

#### Protein identification

Protein identification was carried out at the PAPPSO platform (INRA, Jouy-en-Josas) using MALDI-TOF mass spectrometry (MS). Protein spots were excised from Coomassie blue-stained gels and in-gel digested with trypsin. Gel pieces were placed in Eppendorf tubes and washed with 30 µL 25 mM ammonium carbonate, 50% acetonitrile. The supernatants were discarded and gel pieces were dried at 37°C for 15 min. The gels were rehydrated with 20 µL 50 mM ammonium carbonate containing 100 ng of porcine trypsin (Promega, Madison, WI, USA). The solutions were incubated overnight at 37°C. The supernatants containing peptides were directly analyzed by MALDI-TOF Mass spectrometry on a Voyager DE STR Instrument (Applied Biosystems, Framingham, CA, USA). The α-cyano-4-hydroxycinnamic acid matrix was prepared at 4 mg/mL in 0.1% TFA, 50% acetonitrile. An equal volume (1 µL) of matrix and sample were spotted onto the MALDI-TOF target plate. Spectra were acquired in the reflector mode with the following parameters: 2,000 laser intensity, 20 kV accelerating voltage, 62% grid voltage, 120 ns delay. The mass gates used were 840–3500 Da. Internal calibration was performed by using the trypsin peptides at 842.5 and 2,211.1 Da. Database searches were conducted with the MS-Fit software (http://prospector.ucsf.edu) either on an *L. lactis*-specific protein database.

The few spots which could not be identified by MALDI-TOF were analysed by LC-MS/MS using an Ultimate 3000 LC system (Dionex, Voisins le Bretonneux, France) connected to a linear ion trap mass spectrometer (LTQ, Thermo Fisher, USA) by a nanoelectrospray interface to realize the separation, ionisation and fragmentation of peptides, respectively. The supernatant of trypsin hydrolysis was transferred to a new tube and the gel pieces were extracted with a) 25 µL of buffer B (50 mM ammonium carbonate) and b) two times buffer C (Formic acid 0.1% acetonitrile 50%). For each extraction, the gel pieces were incubated for 15 min at room temperature while shaking. The supernatants of each extraction were pooled with the original trypsin digest supernatants and dried for 2 h in a Speed-Vacuum concentrator. The peptides were then re-suspended in 25 µL of precolumn loading buffer (0.08% TFA and 2% ACN in water). LC-MS/MS analysis was performed on an Ultimate 3000 LC system (Dionex, Voisins le Bretonneux, France) connected to linear ion trap mass spectrometer (LTQ, Thermo Fisher, USA) by nanoelectrospray interface for separation, ionisation and fragmentation of all peptides. Four µL of tryptic peptide mixtures were loaded at flow rate 20 µL/min onto precolumn Pepmap C18 (0.3×5 mm, 100 Å, 3 µm; Dionex). After 4 min, the precolumn was connected with the separating nanocolumn Pepmap C18 (0.075×150 mm, 100 Å, 3 µm, Dionex) and the gradient was started at 300 nL/min. All peptides were separated on the nanocolumn using a linear gradient from 2 to 36% of buffer B, over 18 min. Eluting buffer A: 0.1% Formic acid, 2% acetonitrile and eluting buffer B: 0.1% Formic acid, 80% acetonitrile. Including the regeneration step, each run was 50 min in length. Ionization was performed on liquid junction with a spray voltage of 1.3 KV applied to non-coated capillary probe (PicoTip EMITER 10 µm ID; New Objective, USA). Peptides ions were analysed by the Nth-dependent method as follows: (i) full MS scan (m/z 300–2000), (ii) ZoomScan (scan of the 3 major ions), (iii) MS/MS on these 3 ions with classical peptides fragmentation parameter: Qz = 0.25, activation time = 30 ms, collision energy = 40%. Proteins identifications were performed with Bioworks 3.3 software. The raw data were converted and filtered in peak lists with default data generation parameters for LTQ mass spectrometer. All peak lists of precursor and fragment ions were matched automatically against a *Lactococcus lactis* IL 1403 protein database. The Bioworks search parameter included: trypsin specificity with one missed cleavage, variable oxydation of methionine and the mass tolerance was fixed to 1.4 Da for precursor ion and 0.5 Da for fragment ions. The search results were filtered using Bioworks 3.3. A multiple threshold filter applied at the peptide level consisted of the following criteria: Xcorr magnitude up to 1.7, 2.5 and 3.0 for respectively mono-, di- and tri-charged peptides; peptide probabilities lower than 0.01; ΔCn greater than 0.1 and only the first match result for each identified peptide.

#### Statistical treatment

Raw spot volumes were normalized by the mean intensity of the corresponding gel. A total of 542 spots corresponding to 352 different proteins were detected. Some of the spots corresponded to proteins mixture and were not considered. The intensities of spots corresponding to protein isoforms in a same gel were summed so as to represent the level of a single protein independently of post-transcriptional modifications. 15 proteins identified both on 4.5–5.5 and 5–6 pH ranges displayed very different amounts. We considered that the best protein level estimation was given by the highest signal.

Since total protein concentrations remain stable whatever the growth rate (42±6 g protein per 100 g cell dry weight), the abundance data are considered to be equivalent to concentrations. Ratios were calculated using the slowest growth phase as a reference. The statistical significance of ratios were evaluated using Student test and False Discovery Rates (FDR, calculated according to Benjamini-Hochberg method [51]) calculated with R free statistical software. Proteins with ratio associated to a False Discovery Rate (FDR) lower than 20% were considered as differentially regulated (see Table 1).

### Mathematical treatments

R^2^ calculations and equations resolution were perform with MATLAB software.

### Correlation calculations

Correlations were estimated using R free statistical software to calculate Spearman rank correlation coefficient and the associated p-value.

## Supporting Information

Table S1 Transcriptomic and proteomic raw data and their corresponding standard deviation
(0.24 MB DOC)

Table S2 Regulatory coefficients calculated between the different growth rates
(0.16 MB XLS)

## References

[1] Ideker T, Galitski T, Hood L (2001). A new approach to decoding life: systems biology.. Annu Rev Genomics Hum Genet.

[2] Kitano H (2002). Systems biology: a brief overview.. Science.

[3] Kitano H (2002). Computational systems biology.. Nature.

[4] Williamson MP (2005). Systems biology: will it work?. Biochem Soc Trans.

[5] Joyce AR, Palsson BO (2006). The model organism as a system: integrating ‘omics’ data sets.. Nat Rev Mol Cell Biol.

[6] Greenbaum D, Colangelo C, Williams K, Gerstein M (2003). Comparing protein abundance and mRNA expression levels on a genomic scale.. Genome Biol.

[7] Washburn MP, Koller A, Oshiro G, Ulaszek RR, Plouffe D (2003). Protein pathway and complex clustering of correlated mRNA and protein expression analyses in Saccharomyces cerevisiae.. Proc Natl Acad Sci U S A.

[8] Lu P, Vogel C, Wang R, Yao X, Marcotte EM (2007). Absolute protein expression profiling estimates the relative contributions of transcriptional and translational regulation.. Nat Biotechnol.

[9] Nie L, Wu G, Culley DE, Scholten JC, Zhang W (2007). Integrative analysis of transcriptomic and proteomic data: challenges, solutions and applications.. Crit Rev Biotechnol.

[10] Wu G, Nie L, Zhang W (2008). Integrative analyses of posttranscriptional regulation in the yeast Saccharomyces cerevisiae using transcriptomic and proteomic data.. Curr Microbiol.

[11] Mata J, Marguerat S, Bahler J (2005). Post-transcriptional control of gene expression: a genome-wide perspective.. Trends Biochem Sci.

[12] Brockmann R, Beyer A, Heinisch JJ, Wilhelm T (2007). Posttranscriptional expression regulation: what determines translation rates?. PLoS Comput Biol.

[13] El-Sharoud WM, Graumann PL (2007). Cold shock proteins aid coupling of transcription and translation in bacteria.. Sci Prog.

[14] Kaberdin VR, Blasi U (2006). Translation initiation and the fate of bacterial mRNAs.. FEMS Microbiol Rev.

[15] Proud CG (2007). Signalling to translation: how signal transduction pathways control the protein synthetic machinery.. Biochem J.

[16] Arava Y, Boas FE, Brown PO, Herschlag D (2005). Dissecting eukaryotic translation and its control by ribosome density mapping.. Nucleic Acids Res.

[17] Nie L, Wu G, Brockman FJ, Zhang W (2006). Integrated analysis of transcriptomic and proteomic data of Desulfovibrio vulgaris: zero-inflated Poisson regression models to predict abundance of undetected proteins.. Bioinformatics.

[18] Nie L, Wu G, Zhang W (2006). Correlation of mRNA expression and protein abundance affected by multiple sequence features related to translational efficiency in Desulfovibrio vulgaris: a quantitative analysis.. Genetics.

[19] Belle A, Tanay A, Bitincka L, Shamir R, O'Shea EK (2006). Quantification of protein half-lives in the budding yeast proteome.. Proc Natl Acad Sci U S A.

[20] Pratt JM, Petty J, Riba-Garcia I, Robertson DH, Gaskell SJ (2002). Dynamics of protein turnover, a missing dimension in proteomics.. Mol Cell Proteomics.

[21] Dressaire C, Redon E, Milhem H, Besse P, Loubiere P (2008). Growth rate regulated genes and their wide involvement in the Lactococcus lactis stress responses.. BMC Genomics.

[22] Bolotin A, Wincker P, Mauger S, Jaillon O, Malarme K (2001). The complete genome sequence of the lactic acid bacterium Lactococcus lactis ssp. lactis IL1403.. Genome Res.

[23] Brauer MJ, Huttenhower C, Airoldi EM, Rosenstein R, Matese JC (2008). Coordination of growth rate, cell cycle, stress response, and metabolic activity in yeast.. Mol Biol Cell.

[24] Regenberg B, Grotkjaer T, Winther O, Fausboll A, Akesson M (2006). Growth-rate regulated genes have profound impact on interpretation of transcriptome profiling in Saccharomyces cerevisiae.. Genome Biol.

[25] Calcott PH (1981).

[26] Even S, Lindley ND, Cocaign-Bousquet M (2001). Molecular physiology of sugar catabolism in Lactococcus lactis IL1403.. J Bacteriol.

[27] Even S, Lindley ND, Cocaign-Bousquet M (2003). Transcriptional, translational and metabolic regulation of glycolysis in Lactococcus lactis subsp. cremoris MG 1363 grown in continuous acidic cultures.. Microbiology.

[28] Beyer A, Hollunder J, Nasheuer HP, Wilhelm T (2004). Post-transcriptional expression regulation in the yeast Saccharomyces cerevisiae on a genomic scale.. Mol Cell Proteomics.

[29] Fishov I, Zaritsky A, Grover NB (1995). On microbial states of growth.. Mol Microbiol.

[30] Van Zandt T (2000). How to fit a response time distribution.. Psychon Bull Rev.

[31] Jensen KF, Pedersen S (1990). Metabolic growth rate control in Escherichia coli may be a consequence of subsaturation of the macromolecular biosynthetic apparatus with substrates and catalytic components.. Microbiol Rev.

[32] Goldberg AL, St John AC (1976). Intracellular protein degradation in mammalian and bacterial cells: Part 2.. Annu Rev Biochem.

[33] Kaiser CM, Chang HC, Agashe VR, Lakshmipathy SK, Etchells SA (2006). Real-time observation of trigger factor function on translating ribosomes.. Nature.

[34] Swinger KK, Rice PA (2004). IHF and HU: flexible architects of bent DNA.. Curr Opin Struct Biol.

[35] Kuroda M, Ohta T, Hayashi H (1995). Isolation and the gene cloning of an alkaline shock protein in methicillin resistant Staphylococcus aureus.. Biochem Biophys Res Commun.

[36] Nystrom T, Neidhardt FC (1994). Expression and role of the universal stress protein, UspA, of Escherichia coli during growth arrest.. Mol Microbiol.

[37] Gupta SD, Lee BT, Camakaris J, Wu HC (1995). Identification of cutC and cutF (nlpE) genes involved in copper tolerance in Escherichia coli.. J Bacteriol.

[38] Outten FW, Wood MJ, Munoz FM, Storz G (2003). The SufE protein and the SufBCD complex enhance SufS cysteine desulfurase activity as part of a sulfur transfer pathway for Fe-S cluster assembly in Escherichia coli.. J Biol Chem.

[39] Sharp PM, Li WH (1987). The codon Adaptation Index–a measure of directional synonymous codon usage bias, and its potential applications.. Nucleic Acids Res.

[40] Arava Y, Wang Y, Storey JD, Liu CL, Brown PO (2003). Genome-wide analysis of mRNA translation profiles in Saccharomyces cerevisiae.. Proc Natl Acad Sci U S A.

[41] Daran-Lapujade P, Rossell S, van Gulik WM, Luttik MA, de Groot MJ (2007). The fluxes through glycolytic enzymes in Saccharomyces cerevisiae are predominantly regulated at posttranscriptional levels.. Proc Natl Acad Sci U S A.

[42] ter Kuile BH, Westerhoff HV (2001). Transcriptome meets metabolome: hierarchical and metabolic regulation of the glycolytic pathway.. FEBS Lett.

[43] Tuller T, Kupiec M, Ruppin E (2007). Determinants of protein abundance and translation efficiency in S. cerevisiae.. PLoS Comput Biol.

[44] Lange C, Zaigler A, Hammelmann M, Twellmeyer J, Raddatz G (2007). Genome-wide analysis of growth phase-dependent translational and transcriptional regulation in halophilic archaea.. BMC Genomics.

[45] Wada A (1998). Growth phase coupled modulation of Escherichia coli ribosomes.. Genes Cells.

[46] Vogl T, Brengelmann R, Hinz HJ, Scharf M, Lotzbeyer M (1995). Mechanism of protein stabilization by disulfide bridges: calorimetric unfolding studies on disulfide-deficient mutants of the alpha-amylase inhibitor tendamistat.. J Mol Biol.

[47] Ogura T, Wilkinson AJ (2001). AAA+ superfamily ATPases: common structure–diverse function.. Genes Cells.

[48] Russell JB, Cook GM (1995). Energetics of bacterial growth: balance of anabolic and catabolic reactions.. Microbiol Rev.

[49] Redon E, Loubiere P, Cocaign-Bousquet M (2005). Role of mRNA stability during genome-wide adaptation of Lactococcus lactis to carbon starvation.. J Biol Chem.

[50] Maaløe O, Kjeldgaard NO (1966). Control of macromolecular synthesis..

[51] Benjamini YH, Y (1995). Controlling the false discovery rate: a practical and powerful approach to multiple testing.. J R Statist Soc B:.

