# Supplementary material for: Transcriptome and Proteome Exploration to Model Translation Efficiency and Protein Stability in Lactococcus lactis
Source: PLoS Comput Biol. 2009 Dec 18;5(12):e1000606. doi: 10.1371/journal.pcbi.1000606 (PMC2787624; doi:10.1371/journal.pcbi.1000606)
Supplement: Table S1 — Transcriptomic and proteomic raw data and their corresponding standard deviation (0.24 MB DOC) [file pcbi.1000606.s001.doc]

| Protéine | Prot0.09 |  | Prot0.24 |  | Prot0.47 |  | Prot0.88 |  | mRNA0.09 |  | mRNA0.24 |  | mRNA0.47 |  | mRNA0.88 |  | ARN/prot0.09 | ARN/prot0.24 | ARN/prot0.47 | ARN/prot0.88 |
| --- | --- | --- | --- | --- | --- | --- | --- | --- | --- | --- | --- | --- | --- | --- | --- | --- | --- | --- | --- | --- |
|  | mean value | standard deviation (%) | mean value | standard deviation (%) | mean value | standard deviation (%) | mean value | standard deviation (%) | mean value | standard deviation (%) | mean value | standard deviation (%) | mean value | standard deviation (%) | mean value | standard deviation (%) |  |  |  |  |
| ACCC | 0.002109091 | 11 | 0.002636481 | 12 | 0.002770292 | 2 | 0.001236281 | 11 | 5.323333333 | 14 | 9.896666667 | 21 | 11.47 | 6 | 38.72 | 23 | 2524 | 3754 | 4140 | 31320 |
| ACKA1 | 0.002660156 | 12 | 0.002517543 | 7 | 0.002367012 | 14 | 0.001809929 | 24 | 4.976666667 | 11 | 6.113333333 | 31 | 8.283333333 | 21 | 21.65333333 | 8 | 1871 | 2428 | 3499 | 11964 |
| ACKA2 | 0.003494011 | 19 | 0.001258568 | 11 | 0.001346444 | 8 | 5.98124E-04 | 24 | 3.486666667 | 27 | 3.583333333 | 35 | 4.43 | 6 | 12.40333333 | 20 | 998 | 2847 | 3290 | 20737 |
| ADK | 8.99488E-04 | 14 | 0.001313336 | 7 | 0.001448494 | 12 | 0.002069502 | 24 | 5.373333333 | 6 | 6.056666667 | 27 | 7.533333333 | 15 | 22.23333333 | 9 | 5974 | 4612 | 5201 | 10743 |
| AHPC | 0.019455925 | 8 | 0.014942801 | 13 | 0.011509532 | 9 | 0.003527877 | 19 | 13.63666667 | 21 | 19.86333333 | 23 | 29.96333333 | 13 | 55.15 | 17 | 701 | 1329 | 2603 | 15633 |
| ALS | 0.005497852 | 8 | 0.005850826 | 6 | 0.00605099 | 6 | 0.005369262 | 12 | 23.51333333 | 36 | 33.21333333 | 19 | 50.29333333 | 9 | 135.4133333 | 9 | 4277 | 5677 | 8312 | 25220 |
| ARAT | 6.28450E-04 | 16 | 5.56719E-04 | 26 | 5.65760E-04 | 19 | 2.90207E-04 | 42 | 8.44 | 22 | 14.16333333 | 23 | 23.71333333 | 29 | 26.69666667 | 5 | 13430 | 25441 | 41914 | 91992 |
| ARGS | 0.008365865 | 27 | 0.008415663 | 11 | 0.004260642 | 33 | 0.022751479 | 4 | 6.333333333 | 16 | 9.21 | 27 | 14.03 | 18 | 40.61333333 | 10 | 757 | 1094 | 3293 | 1785 |
| AROH | 0.0017452 | 26 | 0.002794258 | 18 | 0.00364697 | 17 | 0.002389547 | 9 | 7.496666667 | 22 | 16.63333333 | 13 | 23.97333333 | 6 | 50.89 | 6 | 4296 | 5953 | 6573 | 21297 |
| ASPS | 0.001903693 | 21 | 0.002243631 | 19 | 0.002256367 | 14 | 0.00400412 | 11 | 4.516666667 | 25 | 6.316666667 | 22 | 8.876666667 | 20 | 20.7 | 5 | 2373 | 2815 | 3934 | 5170 |
| BCAT | 0.008064027 | 9 | 0.008146271 | 5 | 0.007541567 | 7 | 0.006231889 | 11 | 12.55666667 | 33 | 23.52666667 | 32 | 28.29 | 9 | 56.86333333 | 8 | 1557 | 2888 | 3751 | 9125 |
| CCPA | 0.019005745 | 9 | 0.016551129 | 2 | 0.019898476 | 2 | 0.018086459 | 1 | 6.933333333 | 22 | 9.693333333 | 24 | 11.82 | 7 | 40.98333333 | 16 | 365 | 586 | 594 | 2266 |
| CITE | 0.005078149 | 3 | 0.00608489 | 13 | 0.00599219 | 11 | 0.007715055 | 18 | 11.82333333 | 21 | 18.57333333 | 26 | 26.92666667 | 18 | 30.59 | 7 | 2328 | 3052 | 4494 | 3965 |
| CITF | 0.013452022 | 13 | 0.008116441 | 22 | 0.005122029 | 38 | 0.003930347 | 55 | 9.13 | 23 | 16.05 | 40 | 21.48666667 | 14 | 26.10333333 | 4 | 679 | 1977 | 4195 | 6641 |
| CLPB | 0.001932301 | 14 | 0.002509961 | 19 | 0.001799968 | 2 | 0.001828771 | 10 | 7.48 | 40 | 9.38 | 16 | 13.87 | 9 | 39.84333333 | 10 | 3871 | 3737 | 7706 | 21787 |
| CLPE | 0.003371374 | 17 | 0.002986338 | 14 | 0.001814849 | 5 | 3.47016E-04 | 29 | 4.696666667 | 21 | 5.903333333 | 17 | 8.72 | 11 | 14.24666667 | 22 | 1393 | 1977 | 4805 | 41055 |
| CODY | 0.005360958 | 11 | 0.005294939 | 11 | 0.006020965 | 2 | 0.00346739 | 28 | 10.04666667 | 20 | 13.89333333 | 32 | 20.38 | 7 | 37.8 | 10 | 1874 | 2624 | 3385 | 10902 |
| CSPE | 0.004598923 | 27 | 0.002847717 | 11 | 0.003202612 | 4 | 0.0067417 | 9 | 10.52 | 18 | 15.83333333 | 49 | 20.93 | 32 | 50.17666667 | 34 | 2287 | 5560 | 6535 | 7443 |
| DDL | 0.001885733 | 18 | 0.002058743 | 24 | 0.002445511 | 21 | 0.00156184 | 59 | 3.813333333 | 15 | 5.146666667 | 28 | 6.88 | 10 | 17.33666667 | 9 | 2022 | 2500 | 2813 | 11100 |
| DEF | 7.01976E-04 | 29 | 9.45865E-04 | 6 | 7.62225E-04 | 27 | 1.87025E-04 | 36 | 5.646666667 | 16 | 7.536666667 | 42 | 11.33 | 12 | 13.45333333 | 39 | 8044 | 7968 | 14864 | 71933 |
| DEOD | 0.002348469 | 9 | 0.002445222 | 14 | 0.002623577 | 16 | 0.004559599 | 3 | 6.8 | 8 | 9.273333333 | 30 | 13.22666667 | 15 | 22.85666667 | 30 | 2896 | 3792 | 5041 | 5013 |
| DNAK | 0.016956282 | 18 | 0.022865669 | 4 | 0.033868893 | 9 | 0.029459343 | 7 | 6.36 | 20 | 10.54 | 16 | 14.1 | 7 | 32.38666667 | 8 | 375 | 461 | 416 | 1099 |
| DNAN | 0.007493854 | 5 | 0.009084524 | 15 | 0.008114632 | 9 | 0.009552094 | 17 | 3.213333333 | 17 | 4.773333333 | 24 | 5.686666667 | 28 | 15.2 | 22 | 429 | 525 | 701 | 1591 |
| DPSA | 0.047212116 | 20 | 0.042852015 | 23 | 0.035552247 | 6 | 0.025305975 | 10 | 19.30333333 | 26 | 27.9 | 38 | 38.90333333 | 31 | 68.64333333 | 40 | 409 | 651 | 1094 | 2713 |
| DXSB | 8.84072E-04 | 23 | 8.45002E-04 | 10 | 7.76018E-04 | 15 | 2.53142E-04 | 30 | 2.6 | 21 | 3.863333333 | 22 | 4.593333333 | 7 | 9.24 | 15 | 2941 | 4572 | 5919 | 36501 |
| ENOA | 0.034153889 | 22 | 0.042102623 | 11 | 0.054860182 | 9 | 0.052030093 | 7 | 10.49666667 | 17 | 17.32333333 | 25 | 28.76333333 | 1 | 45.32666667 | 30 | 307 | 411 | 524 | 871 |
| FABF | 0.003471184 | 9 | 0.003287028 | 4 | 0.004008662 | 2 | 0.002410724 | 12 | 2.236666667 | 20 | 4.486666667 | 32 | 5.493333333 | 24 | 11.94 | 11 | 644 | 1365 | 1370 | 4953 |
| FABG1 | 0.002625191 | 18 | 0.002659023 | 15 | 0.00351901 | 17 | 0.010221204 | 60 | 3.97 | 12 | 8.56 | 36 | 10.46333333 | 29 | 24.83333333 | 20 | 1512 | 3219 | 2973 | 2430 |
| FABZ1 | 0.006265698 | 22 | 0.006649592 | 11 | 0.008739825 | 16 | 0.009533866 | 47 | 5.52 | 14 | 8.633333333 | 32 | 10.70333333 | 18 | 17.25666667 | 40 | 881 | 1298 | 1225 | 1810 |
| FBAA | 0.03193441 | 6 | 0.035193272 | 4 | 0.032230489 | 5 | 0.039278805 | 8 | 10.48333333 | 15 | 14.03 | 50 | 19.61333333 | 42 | 33.21 | 28 | 328 | 399 | 609 | 845 |
| FMT | 0.00152446 | 29 | 0.001647315 | 12 | 0.001812475 | 10 | 3.52434E-04 | 61 | 3.786666667 | 15 | 3.513333333 | 39 | 5.093333333 | 23 | 8.53 | 6 | 2484 | 2133 | 2810 | 24203 |
| FRR | 0.00392623 | 10 | 0.006042418 | 3 | 0.007270597 | 11 | 0.01031618 | 9 | 10.29333333 | 25 | 9.883333333 | 30 | 13.74666667 | 5 | 29.29 | 8 | 2622 | 1636 | 1891 | 2839 |
| FTSA | 0.004002629 | 28 | 0.003775924 | 14 | 0.00476061 | 5 | 0.002088444 | 15 | 8.846666667 | 16 | 12.22666667 | 22 | 17.36333333 | 6 | 39.00333333 | 6 | 2210 | 3238 | 3647 | 18676 |
| FTSZ | 0.00353186 | 8 | 0.006476302 | 5 | 0.007633027 | 17 | 0.00161337 | 40 | 5.746666667 | 24 | 8.41 | 24 | 10.38333333 | 9 | 22.32333333 | 2 | 1627 | 1299 | 1360 | 13836 |
| FUSA | 0.013727285 | 26 | 0.015923108 | 10 | 0.022259631 | 11 | 0.031188574 | 22 | 13.82666667 | 15 | 15.79333333 | 39 | 29.27 | 8 | 33.33666667 | 6 | 1007 | 992 | 1315 | 1069 |
| GALE | 6.31582E-04 | 32 | 5.63600E-04 | 21 | 6.63551E-04 | 32 | 2.63210E-04 | 35 | 5.63 | 23 | 6.86 | 29 | 9.1 | 25 | 15.44 | 33 | 8914 | 12172 | 13714 | 58660 |
| GAPA | 0.003904852 | 9 | 0.003690323 | 13 | 0.002862961 | 1 | 0.001157652 | 20 | 3.216666667 | 16 | 4.766666667 | 36 | 6.92 | 17 | 13.43666667 | 26 | 824 | 1292 | 2417 | 11607 |
| GAPB | 0.263284985 | 10 | 0.248657556 | 8 | 0.279543445 | 5 | 0.261754549 | 4 | 10.96333333 | 14 | 19.74333333 | 23 | 28.76 | 18 | 48.50333333 | 32 | 42 | 79 | 103 | 185 |
| GATB | 0.003691955 | 27 | 0.003945814 | 10 | 0.004738692 | 6 | 0.002170492 | 32 | 4.67 | 16 | 6.593333333 | 16 | 9.48 | 6 | 13.48666667 | 22 | 1265 | 1671 | 2001 | 6214 |
| GLK | 2.50574E-04 | 26 | 4.99348E-04 | 23 | 6.09319E-04 | 11 | 6.58580E-04 | 45 | 13.1 | 32 | 12.26666667 | 18 | 16.06666667 | 7 | 30.59333333 | 22 | 52280 | 24565 | 26368 | 46453 |
| GLMS | 6.68982E-04 | 36 | 0.001300839 | 9 | 0.001716418 | 23 | 0.001240573 | 46 | 5.433333333 | 37 | 6.133333333 | 23 | 9.123333333 | 13 | 16.42666667 | 22 | 8122 | 4715 | 5315 | 13241 |
| GLMU | 0.007701791 | 41 | 0.004758652 | 6 | 0.00272756 | 29 | 0.002101318 | 23 | 8.36 | 29 | 9.533333333 | 19 | 12.66 | 12 | 24.43666667 | 6 | 1085 | 2003 | 4642 | 11629 |
| GLTD | 0.004070612 | 29 | 0.002569626 | 27 | 0.002965224 | 16 | 6.61518E-04 | 37 | 8.516666667 | 21 | 13.55 | 22 | 21.54 | 16 | 28.45 | 15 | 2092 | 5273 | 7264 | 43007 |
| GLYA | 0.00410351 | 18 | 0.003509085 | 9 | 0.00440205 | 6 | 0.001166591 | 28 | 9.92 | 39 | 7.393333333 | 6 | 10.43 | 9 | 22.77 | 25 | 2417 | 2107 | 2369 | 19518 |
| GPDA | 0.002219426 | 17 | 0.003069659 | 5 | 0.003089955 | 10 | 7.96103E-04 | 28 | 6.713333333 | 16 | 9.283333333 | 38 | 13.05 | 14 | 22.51 | 15 | 3025 | 3024 | 4223 | 28275 |
| GREA | 0.002677156 | 11 | 0.003137452 | 2 | 0.003496151 | 6 | 0.002688424 | 19 | 3.886666667 | 19 | 4.966666667 | 30 | 5.58 | 24 | 15.11666667 | 16 | 1452 | 1583 | 1596 | 5623 |
| GUAA | 0.004266945 | 33 | 0.005779107 | 7 | 0.007326953 | 20 | 0.004857593 | 14 | 8.16 | 20 | 11.41666667 | 16 | 17.21333333 | 4 | 32.33333333 | 9 | 1912 | 1976 | 2349 | 6656 |
| HMCM | 0.001685671 | 21 | 0.002719479 | 16 | 0.001839843 | 19 | 0.001147436 | 29 | 9.373333333 | 41 | 6.52 | 6 | 9.36 | 14 | 22.05333333 | 29 | 5561 | 2398 | 5087 | 19220 |
| HOM | 2.97274E-04 | 35 | 4.17616E-04 | 7 | 3.92660E-04 | 40 | 2.93492E-04 | 12 | 8.29 | 35 | 6.343333333 | 13 | 8.806666667 | 16 | 19.2 | 24 | 27887 | 15189 | 22428 | 65419 |
| HPT | 0.001136943 | 20 | 0.001523017 | 7 | 0.001609241 | 4 | 0.001505828 | 23 | 5.28 | 14 | 7.8 | 23 | 10.92666667 | 21 | 17.70333333 | 42 | 4644 | 5121 | 6790 | 11757 |
| HSLA | 0.208482165 | 20 | 0.138804455 | 4 | 0.130142744 | 15 | 0.079443246 | 9 | 5.776666667 | 7 | 9.026666667 | 54 | 12.14666667 | 16 | 18.78333333 | 10 | 28 | 65 | 93 | 236 |
| ILVD | 0.011700395 | 27 | 0.017067161 | 13 | 0.016246585 | 11 | 7.61669E-04 | 43 | 4.9 | 15 | 10.93 | 22 | 12.07666667 | 5 | 7.88 | 13 | 419 | 640 | 743 | 10346 |
| LDH | 0.034565002 | 15 | 0.034177357 | 8 | 0.032581605 | 10 | 0.029270764 | 2 | 3.266666667 | 10 | 4.76 | 15 | 6.456666667 | 13 | 14.29333333 | 20 | 95 | 139 | 198 | 488 |
| LEUS | 0.001848141 | 19 | 0.001618317 | 12 | 0.00104687 | 30 | 0.002714817 | 29 | 4.596666667 | 21 | 7.25 | 21 | 9.13 | 6 | 20.51 | 14 | 2487 | 4480 | 8721 | 7555 |
| LLRA | 7.34904E-04 | 13 | 6.20211E-04 | 9 | 5.32351E-04 | 15 | 6.57758E-04 | 25 | 6.53 | 14 | 8.266666667 | 36 | 9.95 | 25 | 17.94333333 | 25 | 8886 | 13329 | 18691 | 27280 |
| LLRC | 0.00764515 | 18 | 0.010136235 | 7 | 0.012251325 | 4 | 0.032940206 | 8 | 8.626666667 | 18 | 11.16666667 | 20 | 15.73666667 | 7 | 39.04333333 | 9 | 1128 | 1102 | 1284 | 1185 |
| LPLL | 4.52522E-04 | 3 | 5.30405E-04 | 21 | 5.55934E-04 | 20 | 5.00952E-04 | 9 | 2.856666667 | 11 | 2.92 | 27 | 3.093333333 | 37 | 8.186666667 | 23 | 6313 | 5505 | 5564 | 16342 |
| LYSA | 7.39595E-04 | 11 | 3.52183E-04 | 8 | 3.81440E-04 | 10 | 0.002160616 | 32 | 4.7 | 25 | 8.913333333 | 27 | 10.83333333 | 23 | 20.91 | 15 | 6355 | 25309 | 28401 | 9678 |
| LYSS | 0.001683508 | 35 | 9.03493E-04 | 29 | 6.84936E-04 | 26 | 0.002640153 | 59 | 5.313333333 | 17 | 8.556666667 | 16 | 10.73666667 | 13 | 28.07 | 10 | 3156 | 9471 | 15675 | 10632 |
| MAE | 0.00993293 | 14 | 0.014819806 | 10 | 0.016074978 | 4 | 0.006122773 | 23 | 9.31 | 16 | 14.47333333 | 41 | 17.24333333 | 23 | 27.84333333 | 4 | 937 | 977 | 1073 | 4548 |
| MENB | 0.002980599 | 15 | 0.003603538 | 6 | 0.004739872 | 10 | 6.75813E-04 | 61 | 7.283333333 | 6 | 10.18666667 | 34 | 13.12333333 | 15 | 20.13333333 | 34 | 2444 | 2827 | 2769 | 29791 |
| METK | 0.00138859 | 9 | 0.00170106 | 5 | 0.001677769 | 9 | 5.68059E-04 | 15 | 5.333333333 | 5 | 7.373333333 | 29 | 13.26 | 6 | 15.24666667 | 20 | 3841 | 4335 | 7903 | 26840 |
| METS | 5.32202E-04 | 38 | 5.73418E-04 | 18 | 3.89082E-04 | 40 | 9.29128E-04 | 61 | 2.593333333 | 15 | 4.286666667 | 18 | 6.586666667 | 10 | 20.97666667 | 19 | 4873 | 7476 | 16929 | 22577 |
| MURC | 0.002666362 | 6 | 0.004356136 | 16 | 0.004254563 | 25 | 0.008393655 | 36 | 5.55 | 8 | 7 | 28 | 10.29666667 | 8 | 20.53333333 | 11 | 2081 | 1607 | 2420 | 2446 |
| MURD | 3.34192E-04 | 10 | 4.97183E-04 | 11 | 4.29438E-04 | 4 | 8.12747E-04 | 24 | 3.616666667 | 8 | 5.16 | 21 | 7.413333333 | 16 | 12.29333333 | 25 | 10822 | 10378 | 17263 | 15126 |
| MURF | 0.00128487 | 26 | 0.001438364 | 8 | 0.001610067 | 12 | 3.31581E-04 | 32 | 3.84 | 19 | 4.086666667 | 14 | 5.59 | 10 | 11.83 | 14 | 2989 | 2841 | 3472 | 35678 |
| NADE | 0.002066727 | 6 | 0.002252381 | 5 | 0.002056969 | 2 | 0.001438901 | 14 | 2.78 | 24 | 4.616666667 | 22 | 6.163333333 | 10 | 11.54333333 | 23 | 1345 | 2050 | 2996 | 8022 |
| NIFS | 0.002067636 | 20 | 0.001281975 | 13 | 0.001888517 | 19 | 0.001103366 | 84 | 3.73 | 19 | 4.726666667 | 17 | 6.356666667 | 19 | 10.83666667 | 41 | 1804 | 3687 | 3366 | 9821 |
| NUSA | 0.005119674 | 11 | 0.004860286 | 4 | 0.005303238 | 14 | 0.004498179 | 18 | 2.703333333 | 16 | 2.73 | 15 | 3.606666667 | 9 | 8.44 | 55 | 528 | 562 | 680 | 1876 |
| PCRA | 0.00115548 | 39 | 0.001405506 | 5 | 7.13288E-04 | 59 | 0.001296783 | 34 | 5.2 | 34 | 9.033333333 | 25 | 10.52666667 | 8 | 21.41333333 | 4 | 4500 | 6427 | 14758 | 16513 |
| PDHA | 0.009177509 | 14 | 0.007762884 | 24 | 0.007236633 | 11 | 0.004339908 | 14 | 4.4 | 24 | 5.59 | 32 | 8.47 | 27 | 17.91 | 29 | 479 | 720 | 1170 | 4127 |
| PDHB | 0.002585585 | 9 | 0.001856961 | 23 | 0.001790141 | 25 | 0.001187617 | 39 | 7.313333333 | 11 | 9.256666667 | 29 | 15.21666667 | 2 | 22.99 | 7 | 2829 | 4985 | 8500 | 19358 |
| PDHD | 0.006756806 | 12 | 0.00480415 | 29 | 0.004156651 | 15 | 0.002319122 | 20 | 4.82 | 14 | 6.456666667 | 26 | 8 | 30 | 13.56 | 18 | 713 | 1344 | 1925 | 5847 |
| PDP | 0.001421657 | 12 | 0.001139839 | 4 | 0.001154962 | 18 | 3.45082E-04 | 28 | 4.413333333 | 24 | 4.863333333 | 30 | 7.173333333 | 4 | 11.47666667 | 33 | 3104 | 4267 | 6211 | 33258 |
| PEPC | 0.008048109 | 19 | 0.007967382 | 5 | 0.008625086 | 10 | 0.001142147 | 54 | 6.793333333 | 24 | 11.32333333 | 33 | 17.64 | 20 | 39.78666667 | 27 | 844 | 1421 | 2045 | 34835 |
| PEPDB | 0.005349891 | 32 | 0.005332744 | 3 | 0.005563166 | 24 | 0.004410871 | 19 | 5.776666667 | 26 | 8.16 | 25 | 11.47666667 | 19 | 21.65333333 | 11 | 1080 | 1530 | 2063 | 4909 |
| PEPN | 0.001507568 | 41 | 8.70175E-04 | 30 | 0.001384639 | 5 | 0.005743562 | 42 | 9.736666667 | 33 | 15.09 | 24 | 18.62666667 | 10 | 38.82 | 22 | 6459 | 17341 | 13452 | 6759 |
| PEPO | 0.007205784 | 14 | 0.005475379 | 15 | 0.005045262 | 2 | 0.002839774 | 16 | 6.64 | 19 | 8.736666667 | 34 | 13.06 | 13 | 17.77666667 | 17 | 921 | 1596 | 2589 | 6260 |
| PEPP | 0.021412979 | 57 | 0.00396499 | 51 | 0.004082358 | 22 | 2.11678E-04 | 81 | 39.92666667 | 18 | 12.24 | 48 | 6.936666667 | 15 | 10.19 | 32 | 1865 | 3087 | 1699 | 48139 |
| PEPT | 0.003475735 | 31 | 0.004373718 | 7 | 0.003996606 | 15 | 0.009254538 | 23 | 18.80666667 | 16 | 29.62333333 | 51 | 49.06666667 | 9 | 40.83333333 | 21 | 5411 | 6773 | 12277 | 4412 |
| PEPV | 0.006246275 | 18 | 0.005984259 | 19 | 0.006061469 | 22 | 0.004632602 | 11 | 2.723333333 | 17 | 3.07 | 43 | 4.143333333 | 32 | 6.97 | 21 | 436 | 513 | 684 | 1505 |
| PFL | 0.00315154 | 11 | 0.00181864 | 16 | 0.00138111 | 17 | 0.001131299 | 17 | 3.956666667 | 23 | 5.246666667 | 22 | 6.203333333 | 21 | 15.64 | 31 | 1255 | 2885 | 4492 | 13825 |
| PGK | 0.035858778 | 7 | 0.03213774 | 10 | 0.030810353 | 5 | 0.024633127 | 14 | 8.163333333 | 14 | 11.33 | 33 | 17.52 | 7 | 32.31666667 | 9 | 228 | 353 | 569 | 1312 |
| PHET | 9.31082E-04 | 19 | 9.57357E-04 | 11 | 0.001412812 | 10 | 0.00155049 | 42 | 3.62 | 20 | 4.556666667 | 28 | 7.333333333 | 15 | 12.77333333 | 40 | 3888 | 4760 | 5191 | 8238 |
| PMG | 0.01962857 | 19 | 0.019745159 | 12 | 0.025633932 | 2 | 0.02542879 | 10 | 7.123333333 | 8 | 10.97333333 | 33 | 16.34333333 | 24 | 27.22666667 | 43 | 363 | 556 | 638 | 1071 |
| PPIB | 0.002577542 | 20 | 0.004601974 | 2 | 0.003306613 | 35 | 0.001951079 | 25 | 16.21333333 | 11 | 22.19 | 48 | 30.30333333 | 33 | 40.01 | 22 | 6290 | 4822 | 9164 | 20507 |
| PRFA | 0.00146657 | 6 | 0.002168923 | 12 | 0.001867863 | 7 | 0.001413654 | 25 | 26.99333333 | 11 | 36.65 | 52 | 62.27333333 | 2 | 60.53666667 | 26 | 18406 | 16898 | 33339 | 42823 |
| PRFC | 9.77272E-04 | 20 | 7.77195E-04 | 19 | 5.58633E-04 | 26 | 7.13667E-04 | 19 | 4.32 | 9 | 4.816666667 | 18 | 6.4 | 9 | 13.36666667 | 27 | 4420 | 6197 | 11457 | 18730 |
| PROS | 0.008592724 | 11 | 0.00582444 | 16 | 0.005348329 | 7 | 0.002847581 | 15 | 12.88666667 | 25 | 13.31 | 20 | 18.34333333 | 15 | 29.10333333 | 3 | 1500 | 2285 | 3430 | 10220 |
| PRSB | 0.003075808 | 11 | 0.003570036 | 7 | 0.005564697 | 4 | 0.00325326 | 38 | 5.963333333 | 19 | 8.673333333 | 18 | 10.89666667 | 21 | 28.01333333 | 12 | 1939 | 2429 | 1958 | 8611 |
| PTNAB | 0.00962568 | 9 | 0.009555699 | 11 | 0.011969013 | 23 | 0.015220872 | 17 | 8.35 | 20 | 13.38 | 13 | 18.41 | 2 | 81.33 | 20 | 867 | 1400 | 1538 | 5343 |
| PTSH | 0.004782661 | 10 | 0.003409978 | 23 | 0.003498176 | 16 | 0.014937486 | 22 | 13.16333333 | 19 | 24.91666667 | 27 | 33.87666667 | 29 | 75.77666667 | 11 | 2752 | 7307 | 9684 | 5073 |
| PTSI | 0.007468492 | 18 | 0.011703742 | 13 | 0.015328463 | 33 | 0.010978931 | 11 | 5.806666667 | 8 | 7.553333333 | 16 | 10.15333333 | 15 | 25.30666667 | 4 | 777 | 645 | 662 | 2305 |
| PTSK | 0.002426057 | 16 | 0.003349536 | 16 | 0.00256394 | 3 | 7.32713E-04 | 40 | 4.096666667 | 9 | 4.57 | 24 | 6.06 | 15 | 11.89 | 3 | 1689 | 1364 | 2364 | 16227 |
| PURB | 0.00351845 | 29 | 0.004890827 | 15 | 0.00321223 | 15 | 0.009507192 | 1 | 6.333333333 | 17 | 8.333333333 | 21 | 11.45666667 | 18 | 26.80333333 | 29 | 1800 | 1704 | 3567 | 2819 |
| PURR | 0.003386396 | 7 | 0.003947315 | 4 | 0.004964523 | 13 | 0.001000201 | 43 | 3.716666667 | 4 | 4.273333333 | 44 | 5.993333333 | 15 | 8.133333333 | 29 | 1098 | 1083 | 1207 | 8132 |
| PYCA | 0.003492495 | 32 | 0.002448601 | 25 | 0.001566575 | 37 | 0.002863431 | 68 | 4.823333333 | 20 | 9.263333333 | 61 | 7.126666667 | 18 | 16.64666667 | 7 | 1381 | 3783 | 4549 | 5814 |
| PYK | 0.071038441 | 7 | 0.1020894 | 4 | 0.09719156 | 7 | 0.095491176 | 2 | 13.23666667 | 7 | 19.56333333 | 22 | 29.68333333 | 6 | 62.17666667 | 17 | 186 | 192 | 305 | 651 |
| PYRB | 0.001115192 | 17 | 0.0014478 | 18 | 0.001336462 | 6 | 0.009001137 | 21 | 4.25 | 12 | 6.483333333 | 24 | 9.33 | 20 | 26.10333333 | 31 | 3811 | 4478 | 6981 | 2900 |
| PYRC | 0.00129139 | 10 | 0.001693069 | 12 | 0.002288222 | 3 | 0.002234934 | 20 | 5.053333333 | 21 | 7.553333333 | 24 | 9.336666667 | 26 | 27.59 | 29 | 3913 | 4461 | 4080 | 12345 |
| PYRE | 2.86953E-04 | 15 | 5.07736E-04 | 13 | 7.52382E-04 | 15 | 0.002246622 | 31 | 6.46 | 19 | 9.893333333 | 38 | 11.85666667 | 11 | 28.8 | 27 | 22512 | 19485 | 15759 | 12819 |
| PYRH | 5.88366E-04 | 10 | 6.57794E-04 | 17 | 8.24215E-04 | 6 | 2.46174E-04 | 44 | 9.276666667 | 11 | 11.90666667 | 24 | 17.58666667 | 9 | 32.28 | 18 | 15767 | 18101 | 21337 | 131127 |
| PYRR | 0.002439635 | 1 | 0.004077833 | 18 | 0.00565034 | 3 | 0.016595685 | 1 | 1.86 | 18 | 1.7 | 26 | 3.093333333 | 19 | 10.43 | 11 | 762 | 417 | 547 | 628 |
| QUEA | 4.54591E-04 | 19 | 4.43070E-04 | 16 | 4.81760E-04 | 21 | 9.18127E-04 | 7 | 6.34 | 7 | 7.636666667 | 29 | 10.47666667 | 11 | 16.45 | 34 | 13947 | 17236 | 21747 | 17917 |
| RECA | 0.005822596 | 25 | 0.008195128 | 17 | 0.005665334 | 11 | 0.006573602 | 6 | 5.923333333 | 16 | 6.573333333 | 23 | 9.1 | 10 | 20.19333333 | 10 | 1017 | 802 | 1606 | 3072 |
| RMLA | 0.00107539 | 27 | 0.001834165 | 3 | 0.002791658 | 17 | 0.002304749 | 22 | 6.27 | 12 | 11.14 | 28 | 16.75666667 | 6 | 38.29666667 | 43 | 5830 | 6074 | 6002 | 16616 |
| RMLB | 0.009675863 | 6 | 0.011077301 | 5 | 0.014601883 | 2 | 0.019857339 | 8 | 6.096666667 | 18 | 9.8 | 16 | 14.28666667 | 9 | 26.07333333 | 43 | 630 | 885 | 978 | 1313 |
| RMLC | 0.001669791 | 13 | 0.001988219 | 6 | 0.001739398 | 7 | 6.19572E-04 | 29 | 10.34333333 | 14 | 16.59 | 25 | 27.03333333 | 5 | 40 | 27 | 6194 | 8344 | 15542 | 64561 |
| RPLA | 0.04026634 | 8 | 0.029373881 | 20 | 0.042828444 | 4 | 0.036155336 | 21 | 7.67 | 19 | 12.59 | 28 | 18.74 | 11 | 35.07 | 37 | 190 | 429 | 438 | 970 |
| RPLC | 0.002796603 | 30 | 0.004021784 | 25 | 0.002145574 | 31 | 0.004865268 | 73 | 0.943333333 | 25 | 0.703333333 | 39 | 1.596666667 | 12 | 2.956666667 | 51 | 337 | 175 | 744 | 608 |
| RPLF | 0.021671451 | 33 | 0.03007156 | 6 | 0.030149238 | 16 | 0.047920884 | 28 | 0.706666667 | 38 | 0.723333333 | 43 | 1.203333333 | 6 | 3.096666667 | 45 | 33 | 24 | 40 | 65 |
| RPLI | 0.019489426 | 19 | 0.016319487 | 12 | 0.013869371 | 13 | 0.007751854 | 38 | 1.92 | 32 | 2.446666667 | 27 | 3.12 | 28 | 11.56666667 | 7 | 99 | 150 | 225 | 1492 |
| RPLJ | 0.002523246 | 33 | 0.007096016 | 7 | 0.007922719 | 7 | 0.009739025 | 7 | 0.756666667 | 50 | 0.923333333 | 14 | 1.683333333 | 26 | 3.27 | 47 | 300 | 130 | 212 | 336 |
| RPLK | 0.027571352 | 7 | 0.029149688 | 6 | 0.03232782 | 9 | 0.024807881 | 23 | 0.846666667 | 33 | 0.8 | 36 | 1.49 | 21 | 3.273333333 | 8 | 31 | 27 | 46 | 132 |
| RPLN | 0.018504143 | 18 | 0.024762929 | 5 | 0.028523159 | 7 | 0.041134965 | 23 | 2.836666667 | 11 | 4.273333333 | 13 | 8.363333333 | 9 | 18.71333333 | 12 | 153 | 173 | 293 | 455 |
| RPLQ | 0.056231524 | 15 | 0.060547802 | 4 | 0.06274576 | 11 | 0.059889911 | 7 | 9.876666667 | 23 | 15.01 | 22 | 23.93666667 | 6 | 58.21 | 10 | 176 | 248 | 381 | 972 |
| RPME | 0.017977867 | 1 | 0.019159315 | 3 | 0.021827766 | 8 | 0.025904241 | 19 | 23.71 | 23 | 43.38333333 | 37 | 64.78666667 | 23 | 95.97 | 34 | 1319 | 2264 | 2968 | 3705 |
| RPOA | 0.003366096 | 11 | 0.003457366 | 6 | 0.004162934 | 1 | 0.004610979 | 15 | 13.95 | 21 | 23.12 | 20 | 36.09 | 17 | 123.6233333 | 11 | 4144 | 6687 | 8669 | 26811 |
| RPSA | 0.027091544 | 3 | 0.032513033 | 0 | 0.033430308 | 4 | 0.048536037 | 7 | 11.05333333 | 9 | 14.81666667 | 38 | 24.53333333 | 9 | 44.16 | 21 | 408 | 456 | 734 | 910 |
| RPSB | 0.007117108 | 29 | 0.006151507 | 29 | 0.00313774 | 34 | 0.014636054 | 17 | 7.67 | 9 | 12.50333333 | 39 | 22.29 | 14 | 39.06666667 | 19 | 1078 | 2033 | 7104 | 2669 |
| RPSC | 0.051265285 | 4 | 0.048817015 | 10 | 0.066586137 | 11 | 0.017928735 | 29 | 5.653333333 | 9 | 8.956666667 | 33 | 15.19666667 | 5 | 38.58666667 | 17 | 110 | 183 | 228 | 2152 |
| RPSD | 0.043045844 | 22 | 0.049483329 | 6 | 0.030610626 | 17 | 0.016170055 | 52 | 5.58 | 12 | 8.273333333 | 53 | 14.05333333 | 18 | 32.17333333 | 46 | 130 | 167 | 459 | 1990 |
| RPSE | 0.036559726 | 27 | 0.045467087 | 16 | 0.036099405 | 18 | 0.049476959 | 23 | 7.433333333 | 22 | 12.01 | 25 | 19.21333333 | 18 | 33.05333333 | 36 | 203 | 264 | 532 | 668 |
| RPSF | 0.001768666 | 22 | 0.002567429 | 4 | 0.003757826 | 7 | 0.003934958 | 29 | 9.196666667 | 5 | 15.9 | 61 | 28.11666667 | 17 | 54.13666667 | 31 | 5200 | 6193 | 7482 | 13758 |
| RPSG | 0.024368747 | 18 | 0.031224819 | 4 | 0.02982954 | 29 | 0.056364381 | 18 | 4.17 | 18 | 5.57 | 20 | 8.476666667 | 5 | 19.98666667 | 29 | 171 | 178 | 284 | 355 |
| RPSH | 0.018166115 | 3 | 0.019292334 | 3 | 0.017549124 | 10 | 0.02046725 | 20 | 7.59 | 16 | 13.18333333 | 18 | 23.12333333 | 9 | 55.8 | 45 | 418 | 683 | 1318 | 2726 |
| RPSJ | 0.03741866 | 10 | 0.043025409 | 18 | 0.043453519 | 9 | 0.016233561 | 43 | 7.866666667 | 25 | 14.78333333 | 16 | 21.78333333 | 11 | 67.87666667 | 18 | 210 | 344 | 501 | 4181 |
| RPST | 0.030090047 | 4 | 0.035791023 | 9 | 0.034873707 | 6 | 0.04221164 | 81 | 6.263333333 | 20 | 10.51 | 32 | 15.33333333 | 20 | 31.91333333 | 10 | 208 | 294 | 440 | 756 |
| SECA | 0.003341645 | 19 | 0.002339173 | 6 | 0.002014304 | 21 | 7.71017E-04 | 42 | 5.533333333 | 37 | 8.543333333 | 27 | 12.50333333 | 26 | 28.20333333 | 17 | 1656 | 3652 | 6207 | 36579 |
| SERS | 0.005783535 | 2 | 0.004307021 | 3 | 0.005674105 | 14 | 0.012074372 | 10 | 4.61 | 12 | 4.81 | 34 | 7.086666667 | 9 | 11.77333333 | 48 | 797 | 1117 | 1249 | 975 |
| SODA | 0.009306515 | 13 | 0.013399662 | 13 | 0.006372811 | 13 | 0.004708805 | 25 | 10.47333333 | 35 | 12.91333333 | 46 | 19.48 | 16 | 30.91666667 | 41 | 1125 | 964 | 3057 | 6566 |
| SSBB | 0.002709783 | 19 | 0.004433119 | 9 | 0.0054216 | 4 | 0.00339083 | 63 | 7.486666667 | 17 | 13.62666667 | 28 | 22.05666667 | 6 | 62.32666667 | 29 | 2763 | 3074 | 4068 | 18381 |
| THIL | 2.63089E-04 | 29 | 3.89148E-04 | 14 | 4.74779E-04 | 31 | 8.03457E-05 | 34 | 3.203333333 | 28 | 3.976666667 | 29 | 6.256666667 | 13 | 16.06666667 | 17 | 12176 | 10219 | 13178 | 199969 |
| THRC | 0.004722562 | 11 | 0.005076443 | 2 | 0.005774735 | 5 | 0.005694198 | 11 | 5.09 | 25 | 7.04 | 34 | 9.476666667 | 13 | 21.68 | 13 | 1078 | 1387 | 1641 | 3807 |
| THYA | 9.46888E-04 | 10 | 0.001069326 | 11 | 0.001068854 | 10 | 2.65960E-04 | 30 | 8.94 | 28 | 12.90333333 | 25 | 17.7 | 10 | 38.50666667 | 7 | 9441 | 12067 | 16560 | 144784 |
| TIG | 0.026536793 | 34 | 0.030491975 | 9 | 0.037363869 | 6 | 0.028708372 | 14 | 6.566666667 | 14 | 8.473333333 | 34 | 12.31 | 23 | 23.15333333 | 45 | 247 | 278 | 329 | 807 |
| TKT | 0.005634508 | 5 | 0.005564359 | 13 | 0.004805085 | 13 | 0.008351422 | 4 | 7.253333333 | 31 | 10.34 | 21 | 15.85 | 9 | 51.52 | 16 | 1287 | 1858 | 3299 | 6169 |
| TPIA | 0.011665132 | 23 | 0.017137078 | 8 | 0.017277405 | 8 | 0.016850327 | 22 | 5.4 | 13 | 8.723333333 | 39 | 12.07333333 | 10 | 18.78333333 | 15 | 463 | 509 | 699 | 1115 |
| TRPS | 0.001189026 | 19 | 0.002106307 | 9 | 0.001590525 | 20 | 3.31397E-04 | 23 | 3.393333333 | 14 | 4.706666667 | 32 | 7.23 | 31 | 12.77 | 41 | 2854 | 2235 | 4546 | 38534 |
| TRXB1 | 0.004124472 | 7 | 0.00426488 | 10 | 0.003722342 | 9 | 7.36381E-04 | 19 | 2.376666667 | 30 | 3.453333333 | 17 | 4.76 | 16 | 7.243333333 | 12 | 576 | 810 | 1279 | 9836 |
| TSF | 0.014585079 | 3 | 0.016897937 | 7 | 0.020074348 | 4 | 0.02510699 | 0 | 4.236666667 | 22 | 8.593333333 | 26 | 12.81333333 | 11 | 31.40333333 | 11 | 290 | 509 | 638 | 1251 |
| TYPA | 0.003503525 | 12 | 0.003898485 | 4 | 0.00455935 | 6 | 0.006790169 | 15 | 8.5 | 32 | 14.09 | 18 | 20.10333333 | 16 | 42.42 | 15 | 2426 | 3614 | 4409 | 6247 |
| TYRS | 0.011153539 | 14 | 0.016177749 | 10 | 0.015461844 | 5 | 0.026218677 | 5 | 3.623333333 | 19 | 6.523333333 | 23 | 9.846666667 | 3 | 24.46 | 21 | 325 | 403 | 637 | 933 |
| YAHB | 0.006275196 | 24 | 0.003739692 | 22 | 0.002393581 | 7 | 5.41640E-04 | 31 | 6.166666667 | 24 | 6.743333333 | 34 | 9.406666667 | 34 | 13.63333333 | 43 | 983 | 1803 | 3930 | 25170 |
| YAHG | 0.001360869 | 16 | 0.001265945 | 4 | 0.002012776 | 31 | 0.001529042 | 81 | 6.696666667 | 22 | 9.64 | 15 | 13.90333333 | 11 | 26.97333333 | 22 | 4921 | 7615 | 6908 | 17641 |
| YBJJ | 0.002780314 | 38 | 0.003272751 | 4 | 0.004177059 | 20 | 4.36301E-04 | 68 | 6.49 | 20 | 7.333333333 | 13 | 10.51 | 6 | 19.29 | 12 | 2334 | 2241 | 2516 | 44213 |
| YCGE | 0.001312706 | 36 | 0.001880317 | 23 | 0.001322774 | 26 | 0.001178976 | 28 | 4.17 | 12 | 4.916666667 | 24 | 6.393333333 | 10 | 12.55333333 | 22 | 3177 | 2615 | 4833 | 10648 |
| YCIC | 9.70343E-04 | 12 | 0.001183753 | 1 | 0.001332452 | 3 | 0.001632707 | 61 | 3.206666667 | 13 | 3.886666667 | 22 | 5.27 | 12 | 15.27 | 5 | 3305 | 3283 | 3955 | 9353 |
| YDJD | 5.64772E-04 | 22 | 6.59534E-04 | 26 | 7.08535E-04 | 4 | 2.95957E-04 | 8 | 2.916666667 | 11 | 3.706666667 | 23 | 5.12 | 3 | 11.57666667 | 15 | 5164 | 5620 | 7226 | 39116 |
| YEIG | 0.001812291 | 9 | 0.00207424 | 13 | 0.001635053 | 8 | 1.56646E-04 | 28 | 4.45 | 9 | 6.963333333 | 40 | 11.01 | 23 | 24.67666667 | 13 | 2455 | 3357 | 6734 | 157532 |
| YNGE | 0.002133512 | 17 | 0.001961631 | 13 | 0.00238508 | 11 | 0.001766781 | 66 | 4.233333333 | 24 | 6.13 | 21 | 7.96 | 12 | 36.17666667 | 18 | 1984 | 3125 | 3337 | 20476 |
| YNIH | 0.00196026 | 10 | 0.001628204 | 18 | 0.001819582 | 14 | 0.001624667 | 9 | 7.693333333 | 27 | 12.35 | 16 | 12.02666667 | 19 | 30.36666667 | 10 | 3925 | 7585 | 6610 | 18691 |
| YPDB | 0.005011539 | 8 | 0.002513066 | 10 | 0.002876651 | 32 | 0.003791859 | 60 | 6.136666667 | 16 | 7.92 | 33 | 7.906666667 | 4 | 33.14 | 8 | 1225 | 3152 | 2749 | 8740 |
| YPDC | 0.002663073 | 13 | 0.001299877 | 30 | 0.001128232 | 15 | 0.001347018 | 37 | 7.62 | 10 | 8.073333333 | 40 | 8.4 | 18 | 43.59666667 | 10 | 2861 | 6211 | 7445 | 32365 |
| YPDD | 0.027682885 | 12 | 0.02892259 | 5 | 0.018813196 | 13 | 0.019060566 | 2 | 22.14333333 | 37 | 28.80666667 | 13 | 39.33 | 13 | 133.7466667 | 19 | 800 | 996 | 2091 | 7017 |
| YPJH | 0.00765984 | 14 | 0.016779713 | 11 | 0.016818049 | 8 | 5.96164E-04 | 67 | 4.923333333 | 21 | 9.483333333 | 32 | 14.28666667 | 2 | 24.61666667 | 15 | 643 | 565 | 849 | 41292 |
| YRAB | 0.003717882 | 28 | 0.006997123 | 5 | 0.00616907 | 18 | 0.002485459 | 10 | 2.56 | 35 | 3.293333333 | 44 | 4.796666667 | 8 | 9.95 | 20 | 689 | 471 | 778 | 4003 |
| YRBA | 8.41622E-04 | 23 | 6.23846E-04 | 3 | 6.13091E-04 | 5 | 9.77549E-04 | 20 | 6.59 | 25 | 8.846666667 | 26 | 14.03666667 | 12 | 32.02 | 2 | 7830 | 14181 | 22895 | 32755 |
| YRCA | 0.001368114 | 36 | 7.01667E-04 | 3 | 7.50651E-04 | 13 | 0.001622346 | 31 | 5.343333333 | 27 | 6.043333333 | 28 | 7.6 | 4 | 33.96666667 | 8 | 3906 | 8613 | 10125 | 20937 |
| YSEF | 0.001396073 | 2 | 0.001417244 | 12 | 0.001083521 | 3 | 3.07165E-04 | 29 | 4.293333333 | 10 | 7.43 | 39 | 9.65 | 15 | 16.80333333 | 15 | 3075 | 5243 | 8906 | 54705 |
| YSFB | 9.44484E-04 | 7 | 0.001225303 | 2 | 0.001483488 | 3 | 5.75544E-04 | 77 | 3.62 | 15 | 5.863333333 | 49 | 7.88 | 28 | 10.99 | 27 | 3833 | 4785 | 5312 | 19095 |
| YTAA | 0.004872012 | 24 | 0.004632694 | 4 | 0.003088173 | 20 | 3.16070E-04 | 50 | 5.72 | 8 | 6.863333333 | 30 | 10.31 | 22 | 18.28 | 12 | 1174 | 1481 | 3339 | 57835 |
| YTDB | 0.006099738 | 11 | 0.00697455 | 10 | 0.00567852 | 16 | 0.003190867 | 30 | 19.48333333 | 16 | 28.87 | 33 | 43.22 | 7 | 64.82333333 | 7 | 3194 | 4139 | 7611 | 20315 |
| YTGG | 9.67713E-04 | 24 | 9.93615E-04 | 6 | 8.07249E-04 | 6 | 2.48552E-04 | 35 | 12.82666667 | 5 | 17.49 | 20 | 30.83 | 1 | 29.6 | 20 | 13255 | 17602 | 38191 | 119090 |
| YTGH | 0.018489453 | 25 | 0.00870781 | 18 | 0.006265293 | 27 | 5.63058E-04 | 36 | 15.97333333 | 26 | 15.17333333 | 28 | 20.14666667 | 17 | 36.73666667 | 5 | 864 | 1742 | 3216 | 65245 |
| YTHC | 0.007941584 | 27 | 0.011943096 | 44 | 0.007790649 | 21 | 0.003168854 | 6 | 5.913333333 | 24 | 13.29333333 | 23 | 10.61666667 | 24 | 38.88 | 12 | 745 | 1113 | 1363 | 12269 |
| YTJH | 0.001382017 | 15 | 0.001030213 | 10 | 0.001165505 | 19 | 0.001116723 | 21 | 6.113333333 | 12 | 8.54 | 22 | 12.80666667 | 5 | 26.77333333 | 14 | 4423 | 8290 | 10988 | 23975 |
| YUHE | 0.001081114 | 27 | 0.00251337 | 17 | 0.003156288 | 35 | 6.49721E-04 | 41 | 10.02 | 24 | 13.56666667 | 21 | 18.49666667 | 12 | 47.25 | 14 | 9268 | 5398 | 5860 | 72723 |
| YWCC | 0.01497224 | 15 | 0.005485898 | 5 | 0.006734067 | 10 | 0.005364913 | 65 | 7.683333333 | 20 | 7.516666667 | 17 | 9.84 | 13 | 37.56666667 | 21 | 513 | 1370 | 1461 | 7002 |
| YWED | 0.001103892 | 12 | 0.001363611 | 16 | 0.001057552 | 15 | 1.29624E-04 | 23 | 2.49 | 2 | 2.436666667 | 12 | 3.206666667 | 11 | 7.036666667 | 9 | 2256 | 1787 | 3032 | 54285 |
| ZWF | 0.006860923 | 17 | 0.007414541 | 8 | 0.006579851 | 8 | 0.009944694 | 63 | 4.963333333 | 11 | 5.993333333 | 22 | 7.936666667 | 13 | 23.42 | 2 | 723 | 808 | 1206 | 2355 |
|  |  |  |  |  |  |  |  |  |  |  |  |  |  |  |  |  |  |  |  |  |
|  |  |  |  |  |  |  |  |  |  |  |  |  |  |  |  |  | 0 | 0 | 0 | 1 |
|  |  |  |  |  |  |  |  |  |  |  |  |  |  |  |  |  | 0 | 0 | 0 | 1 |
